# Color-Stable Formulations for 3D-Photoprintable Dental Materials

**DOI:** 10.3390/polym16162323

**Published:** 2024-08-16

**Authors:** David Bassenheim, Kai Rist, Norbert Moszner, Yohann Catel, Robert Liska, Patrick Knaack

**Affiliations:** 1 Institute of Applied Synthetic Chemistry, Vienna University of Technology, Getreidemarkt 9/163 MC, A-1060 Vienna, Austria; 2 Ivoclar Vivadent AG, Bendererstrasse 2, FL-9494 Schaan, Liechtensteinyohann.catel@ivoclar.com (Y.C.)

**Keywords:** color stability, photoinitiator, light curing, 3D printing, dental material, TPO, UDMA

## Abstract

Color stability is crucial for dental materials to ensure they perfectly match a patient’s tooth color. This is particularly challenging in photoresist-based additive manufacturing. Although some studies have addressed this issue, the exact causes of discoloration and ways to minimize it remain unclear. In this study, the intrinsic causes of discoloration in materials intended for 3D printing are investigated by examining thin-film samples (1200 µm) of various compositions, which are stored under different conditions. The samples are evaluated by measuring the UV-Vis absorption spectra at regular intervals to monitor changes. The findings reveal that both the composition of the formulations and the storage conditions significantly influence the discoloration behavior. Furthermore, methods have been developed to reduce or completely prevent discoloration. The use of photoinitiators with sterically demanding benzoyl moieties, as well as the addition of stabilizers, effectively decreases the intensity of emerging discoloration. Furthermore, incorporating the oxidizing agent cumene hydroperoxide (CHP) results in materials that maintain color stability.

## 1. Introduction

Additive manufacturing is a technology that is becoming increasingly important in dentistry [1,2,3,4]. It offers the possibility of producing complex structures with minimal waste and low costs [5]. The diverse application possibilities are described in various publications [6,7,8,9]. A widely used method of 3D printing in the dental field is digital light processing (DLP) [10,11,12,13,14]. In this technique, a photoresist is cured by illumination with a projector, creating the desired 3D object layer by layer. The crucial components of a formulation for DLP printing are the photoinitiator and the monomer. The monomers used in dentistry for this application are mostly methacrylates, with 1,6-bis-[2-methacryloyloxyethoxycarbonylamino]-2,4,4-trimethylhexane (UDMA) being one of the most important [10,15,16,17,18,19]. With regard to the photoinitiator, it is important to note that most DLP printers use projection systems emitting light at 385 nm or 405 nm [20]. For this reason, acylphosphine oxides, such as 2,4,6-trimethylbenzoyldiphenylphosphine oxide (TPO) or bis (2,4,6-trimethylbenzoyl)-phenylphosphineoxide (BAPO), are ideal photoinitiators, as they are highly efficient at these wavelengths [4,20,21,22]. From an aesthetic point of view, the color stability of a dental restorative is essential, but especially with 3D-printed materials, some works show that this color stability is not a given [22,23,24,25]. With regard to intrinsic factors, the literature shows that, particularly, the monomer and the photoinitiator have a significant influence on the discoloration behavior [13,26,27,28,29,30,31,32,33,34,35,36]. Related to the monomer, a correlation was found between water absorption, water solubility and color stability of the polymers produced [28]. Unreacted double bonds of the monomer are also held responsible for the discoloration [27]. In the case of photoinitiators, high initiator concentrations and the associated residual photoinitiators appear to be one possible cause of discoloration [33,34,35]. On the other hand, different photoinitiators seem to cause different discolorations, which could be attributed to the formed photolysis products [36,37]. For acylphosphine oxides, mesitaldehyde, mesitil, or mesitoin could be such colored photolysis products. In the work of Griesser et al., the formation of mesitaldehyde and mesitil was actually demonstrated in the case of the photoinitiator BAPO [38]. Since TPO has been proven to exhibit better color stability than BAPO [30,31,32,39], it makes sense that TPO is the most commonly used photoinitiator for 3D printing in the dental sector [4,10,11,14,22,40,41,42].

The aim of this work is, as a first step, to understand the mechanisms that are responsible for the discoloration of cured TPO-based materials. For this purpose, the influence of the cured sample storage conditions (temperature, dry/wet, and with/without oxygen), as well as the nature of the formulation (nature of the monomer/photoinitiator and addition of various stabilizers and an oxidizing agent), on the color stability were studied. The findings of this investigation will then be used to develop methods to minimize or even completely prevent discoloration. The modification of the photoinitiators used and the addition of additives should prove to be suitable options. 

## 2. Materials and Methods

### 2.1. Chemicals

The photoinitiator 2,4,6-trimethylbenzoyldiphenylphosphine oxide (TPO) was provided as a gift from Lambson (Leeds, West Yorkshire), bis (2,4,6-trimethylbenzoyl)-phenylphosphineoxide (BAPO) from BASF (Ludwigshafen, Germany), 1-hydroxy-cyclohexyl-phenyl-ketone (I184) from Ciba-Geigy (Basel, Switzerland), 2-hydroxy-4’-(2-hydroxyethoxy)-2-methylpropiophenon (I2959) from Ciba (Basel, Switzerland), 2-hydroxy-2-methylpropiophenon (D1173) from Merck (Darmstadt, Germany), and bis(4-methoxybenzoyl)diethylgermane (Ivocerin^®^) from Ivoclar (Schaan, Liechtenstein). The photoinitiators MAPO-1 and MAPO-2 were synthesized by Ivoclar (Schaan, Liechtenstein) as described in the literature [43]. The monomers 1,6-bis-[2-methacryloyloxyethoxycarbonylamino]-2,4,4-trimethylhexane (UDMA, mixture of isomers) and decandiol-1,10-dimethacrylate (D_3_MA, stabilized with 20 ppm BHT) were kindly provided by Ivoclar (Schaan, Liechtenstein). The stabilizers 3,5-di-*tert*-4-butylhydroxytoluol (BHT), *tert*-butylhydroquinone (TBHQ), and (±)-α-tocopherol (TP) were purchased from Aldrich (Milwaukee, WI, USA), pentaerythritol tetrakis [3-(3,5-di-*tert*-butyl-4-hydroxyphenyl)propionate] (PTP) from TCI (Tokyo, Japan), pyrogallol (PyG) from Merck (Darmstadt, Germany), tris (4-*tert*-butyl-3-hydroxy-2,6-dimethylbenzyl)isocyanurate (TIC) from Santa Cruz (Dallas, TX, USA), and 4-methoxyphenol (MeHQ) and propyl gallate (PrG) from Fluka (Morris Plains, NJ, USA). 2,4,6-Trimethylbenzaldehyde (mesitaldehyde), 4,4’-dimethoxybenzoin (*p*-anisoin), 4,4’-dimethoxybenzil (*p*-anisil), 4-methoxybenzaldehyde (anisaldehyde), and cumene hydroperoxide (CHP, 80% solution in cumene) were purchased from Aldrich (Burlington, MA, USA), 2-hydroxy-1,2-diphenylethanon (benzoin) from Merck (Darmstadt, Germany), 1,2-diphenylethan-1,2-dion (benzil) from ACROS (Geel, Belgium), and benzaldehyde from Carl Roth (Karlsruhe, Germany). All chemicals were used as received unless otherwise noted.

### 2.2. Color Stability and Sample Preparation

Two methods were chosen to evaluate the color stability. On the one hand, cured polymer samples and references were analyzed by UV-Vis absorption measurements. The shapes of the absorption bands provide relevant information about the discoloration process. On the other hand, analysis was carried out using the CIELAB color space in order to quantify the actual discoloration and evaluate the color.

In each individual experiment, all samples were prepared and measured in one set to ensure comparability. The respective samples were analyzed by means of single measurements.

#### 2.2.1. UV-Vis

For the measurements, formulations had to be prepared. Unless otherwise stated, the standard formulation consisted of the monomer UDMA (stabilized with 100 ppm MeHQ) and 1.1 mol% of the respective photoinitiator. The formulations were placed in an ultrasonic bath at 50 °C for 10 min, resulting in a notable reduction in viscosity. The formulations were then homogenized for a period of 5 min using a vortex mixer. This process was repeated alternately until the complete dissolution of all components was achieved.

Thin-layer polymer samples were prepared from the formulations for measuring the absorption spectra. In order to quantify the change in absorption, it is essential that all samples have exactly the same layer thicknesses. These exact layer thicknesses were achieved by preparing the samples using a photorheometer (MCR302, Anton Paar, Graz, Austria) [44]. Just like in a usual rheometer, a certain gap width can be set between the flat stamp and the plate. In the photorheometer, the plate is made of quartz glass and has a beam path underneath, which can be coupled with a light source. The first experiments with different layer thicknesses show that 1200 µm is a suitable thickness for measuring and evaluating discolorations. Depending on the photoinitiator in the respective sample, different light sources were used for curing. The TPO- and BAPO-based samples were irradiated with an OmniCure LX500 LED Spot UV Curing System (Excelitas Technologies Corp., Pittsburgh, PA, USA) with a 385 nm LED head. An OmniCure S2000 Spot UV curing system with a medium-pressure mercury lamp (Excelitas Technologies Corp., Pittsburgh, PA, USA) was used together with a 400-500 nm filter for curing Ivocerin^®^-based samples and with a 320-500 nm filter for curing I2959, D1173- and I184-based samples. The light intensity at the glass surface of the photorheometer was set to 10 mW/cm^2^ for all samples, and the irradiation time was 300 s. Afterwards, the samples were postcured either with an Ivoclar PrograPrint^®^ Cure device (405 nm/460 nm; program: “Model”; and 270 mW/cm^2^) (Ivoclar, Schaan, Liechtenstein) for TPO-, BAPO-, and Ivocerin^®^-based samples or with an Uvitron IntelliRay 600 UV flood curing system (broadband, 15 min; UV-A: 125 mW/cm^2^, Vis: 125 mW/cm^2^) (Uvitron International, Inc., West Springfield, MA, USA) in the cases of I2959, D1173, and I184.

The absorption spectra were recorded using a Thermo Scientific™ NanoDrop™ One spectrophotometer (Thermo Fisher Scientific Inc., Waltham, MA, USA). The thin-film samples were cut into pieces (≈9 mm × 25 mm) and placed upright in a 10 mm × 10 mm quartz cuvette, and liquid samples were measured as solutions in acetonitrile. The samples were usually stored under light exclusion and dry conditions at 37 °C. For samples with different storage conditions, this will be noted separately.

**Figure 1:**
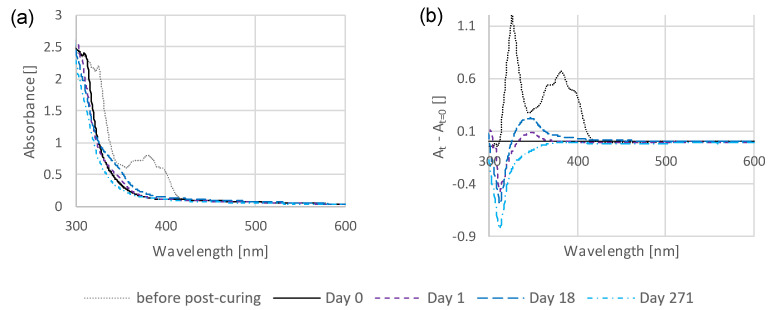
Absorption spectra of a TPO-based thin-film sample at different times (**a**) and the same measurements shown as the difference from day 0 (**b**).

**Figure 2:**
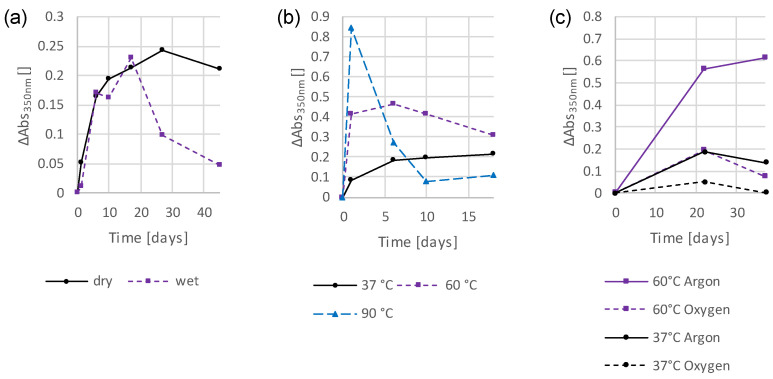
Temporal changes in absorbance at 350 nm of TPO-based thin-film samples under different storage conditions: dry and wet storage at 37 °C (**a**); dry storage at different temperatures (**b**); dry storage in the presence and absence of oxygen (**c**).

Evaluation of UV-Vis measurements:

Figure 1a shows the absorption spectra of a TPO-based standard sample from before postcuring up to 271 days of storage. To better emphasize the temporal difference in the measurements, the absorption spectrum of day 0 can be subtracted from the remaining spectra. This produces the spectra shown in Figure 1b, which can be recognized by the label on the y-axis. In order to compare many different samples with each other, a different form of visualization was chosen. Here, the change in absorbance at the wavelength of 350 nm is plotted against time. Such a diagram can be seen in Figure 2, for example, and can also be recognized by the y-axis.

#### 2.2.2. Cielab

For the measurements in the CIELAB color space, formulations were prepared which were then homogenized with a magnetic stirrer. The composition of the formulations is indicated along with the respective measurements. The test specimens were produced in a steel-ring mold (Ø = 20 mm, H = 2 mm) and cured on both sides using the PrograPrint^®^ Cure device (405 nm/460 nm; program: “Splint”) (Ivoclar, Schaan, Liechtenstein). The samples were immersed in water and stored at 50 °C. The measurements were performed using a CM3700d (Konica Minolta Sensing Americas, Inc., Ramsey, NJ, USA) spectrophotometer in transmitted light against black and white reference surfaces.

The CIELAB color space expresses colors using 3 axes, of which L* indicates the lightness from 0 (black) to 100 (white), a* the green–red axis (−a: green; +a: red), and b* the blue–yellow axis (−a: blue; +a: yellow). The color changes (ΔE) were evaluated by the following Equation (1):

(1) $$∆E=\sqrt{{∆L}^{2}+{∆a}^{2}+{∆b}^{2}}$$

## 3. Results

### 3.1. Time-Dependent Discoloration and Bleaching

For the initial tests, a thin-film sample, consisting of UDMA (stabilized with 100 ppm MeHQ) and the standard photoinitiator TPO (1.1 mol%), was prepared by photo curing in the photorheometer (MCR302, Anton Paar, Graz, Austria). Subsequently, the sample was stored for a total of 271 days under standard conditions (dry at 37 °C). Figure 1a shows the measured absorption spectra at different points in time and Figure 1b the same spectra, with the absorption of day 0 subtracted.

Without any postcuring, the typical absorbance of TPO, between 350 and 420 nm, can be seen, disappearing completely after the postcuring. The absorption spectrum of the TPO is shown in Figure S14a for comparison. The curves at day 1 and day 18 show a developing absorbance, particularly in the range of 320-420 nm. The curve at day 271, on the other hand, even shows a lower absorbance in this range than day 0. Therefore, an absorbance band develops first, which can cause discoloration that disappears over a longer period of time.

### 3.2. Preliminary Investigations into the Influence of Storage Conditions and Formulation Components on the Discoloration Behavior

Subsequently, different storage conditions were tested and the changes in absorbance were plotted against time in graphs to highlight the differences. The absorbance at 350 nm was chosen for this analysis as it demonstrates significant changes, allowing for a reliable evaluation. Figure 2a shows the differences between a sample stored under dry conditions and one stored under wet conditions, immersed in water. Here it can be seen that initially both samples behaved similarly. However, with prolonged storage, the wet-stored sample bleached quickly from day 17. The absorbance of the dry stored sample, on the other hand, continued to increase and subsequent bleaching started later. Figure 2b compares different storage temperatures. If the sample was stored at 37 °C, it can be seen that the absorbance rose relatively flatly but steadily up to the last measuring point (day 18). At 60 °C, a steeper increase can already be observed, with a maximum being reached after 6 days. The trend then reversed and the absorbance decreased. If the sample was stored at 90 °C, the process accelerated further and the maximum was reached after one day of storage. After 10 days, the subsequent decolorization process appeared to be complete, and the absorbance did not decrease any further. In Figure 2c, the influence of oxygen was investigated. For this purpose, one sample was stored in an oxygen atmosphere, while another was stored in an argon atmosphere, thus excluding oxygen. For both the 37 °C and at 60 °C storage temperatures, it can be seen that the exclusion of oxygen led to higher absorbances and, thus, stronger discolorations of the samples. Even at a storage temperature of 37 °C, this can be seen with the naked eye (Figure S3d), although the discoloration is generally barely visible with the low layer thicknesses used. At 60 °C, this effect is noticeably more pronounced, as the photograph in Figure S3e illustrates. The subsequent bleaching of the samples also seemed to take place faster with the oxygen-stored samples.

The corresponding absorption spectra for Figure 2 are provided in the supporting information in Figures S1–S3.

**Figure 3:**
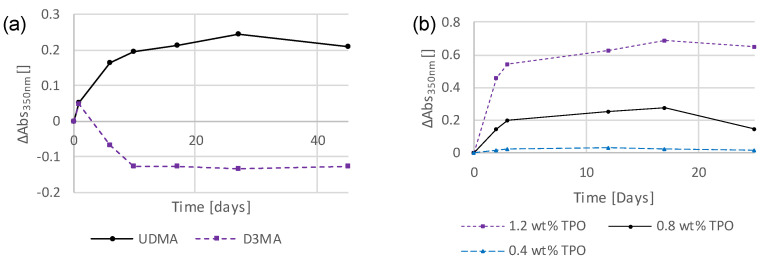
Temporal changes in absorbance at 350 nm of thin-film samples consisting of (**a**) UDMA (stabilized with 100 ppm MeHQ) and TPO (1.1 mol%), and D3MA (stabilized with 20 ppm BHT) and TPO (1.1 mol%); (**b**) UDMA (stabilized with 100 ppm MeHQ) and TPO (0.4 wt% ≙ 0.54 mol%; 0.8 wt% ≙ 1.1 mol%; 1.2 wt% ≙ 1.62 mol%).

Next, a standard UDMA (stabilized with 100 ppm MeHQ) sample was compared with a sample based on a different monomer. The thin-film sample containing D3MA and the photoinitiator TPO shows similar absorption bands to the UDMA-based ones (Figure S4). However, the intensity and persistence of the observed absorbance were significantly lower in the D3MA-based sample (Figure 3a). This shows that the polymer matrix has an effect on the quantity, but not the quality of the discoloration. This demonstrates that the polymer matrix has no qualitative influence on the discoloration but can make a considerable difference in terms of quantity.

Figure S5 shows the discoloration behavior of a thin-film sample based on UDMA previously purified by column chromatography compared to nontreated UDMA (stabilized with 100 ppm MeHQ). This experiment shows that the sample with purified UDMA developed significantly stronger absorbances after just a few days, leading to strong discoloration in comparison to the standard sample. In this case, the separation of the stabilizer seems to have a negative effect on the color stability of the sample.

Next, different concentrations of the photoinitiator TPO (0.4 wt% ≙ 0.54 mol%, 0.8 wt% ≙ 1.1 mol%, and 1.2 wt% ≙ 1.62 mol%) in UDMA (stabilized with 100 ppm MeHQ)-based thin-film samples were tested. Here, it is clearly shown that higher initiator contents result in stronger absorptions and, thus, stronger discolorations (Figure 3b), but the shapes of these absorbances were the same in all cases, only more or less pronounced (Figure S6).

**Figure 4:**
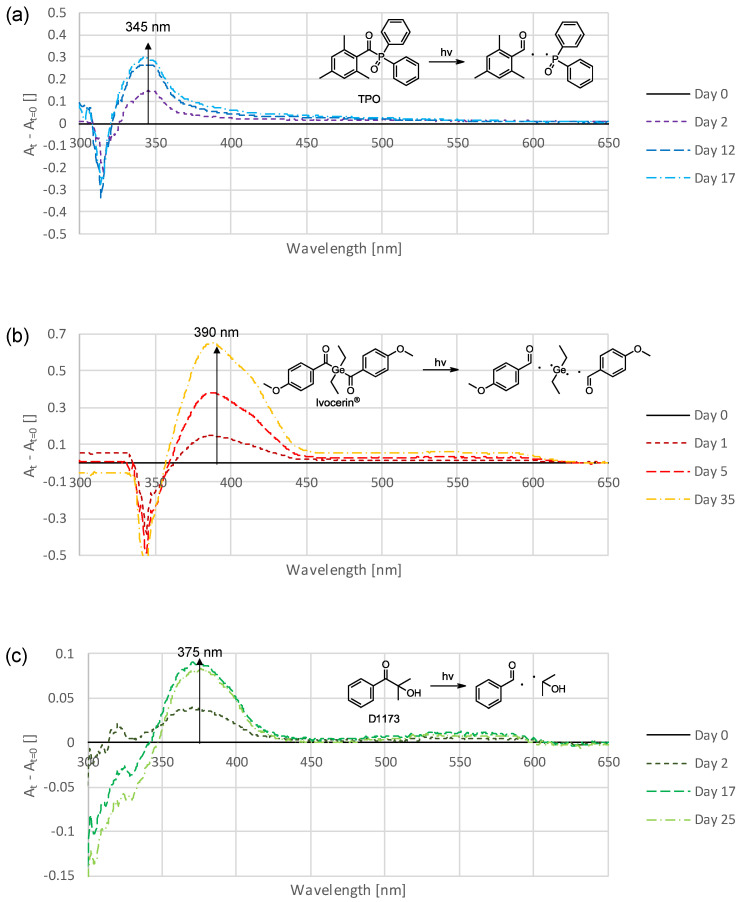
Time-dependent difference spectra of the absorption of thin-film samples consisting of UDMA (stabilized with 100 ppm MeHQ) and 1.1 mol% of different photoinitiators: TPO (**a**); Ivocerin^®^ (**b**); D1173 (**c**).

Subsequently, the effects of different photoinitiators were investigated. Three substances were selected which differed considerably in terms of their structures, absorptions, and the radicals formed. The phosphorus-based photoinitiator TPO was compared with the germanium-based Ivocerin^®^ and the carbon-based Darocur 1173 (D1173). Figure 4 shows the absorption spectra of the thin-film samples formulated with 1.1 mol% of the respective photoinitiator and UDMA (stabilized with 100 ppm MeHQ).

These diagrams show that each initiator caused differences in the emerging absorbances. While TPO (Figure 4a) showed a maximum increase in absorbance at approximately 345 nm, for Ivocerin^®^ (Figure 4b) this was at 390 nm and for D1173 (Figure 4c) at 375 nm. Furthermore, the weak absorbances at higher wavelengths must also be mentioned. For TPO, increasing absorbances can also be observed at around 385 nm and continuously up to 550 nm. In the case of Ivocerin^®^, this was the case between 450 and 625 nm and for D1173 in the range of 450 – 600 nm. In previous experiments, the shift and shape of the rising absorption did not change, but here it is shown that they were influenced by the nature of the photoinitiator. It therefore appears that the structures of the photoinitiators cause quantitative differences in the resulting discolorations.

In Figure S7, these photoinitiators are compared with others that have similar benzoyl chromophores, namely, TPO with BAPO, Ivocerin^®^ with I2959 (2.2 mol%), and D1173 with I184. This diagram shows that the resulting absorbances were qualitatively the same for initiators with similar benzoyl groups. Therefore, modifications to the benzoyl chromophore of photoinitiators could be used to suppress emerging absorbances or to shift them into a nonvisible region.

### 3.3. Photoinitiators

**Figure 5:**
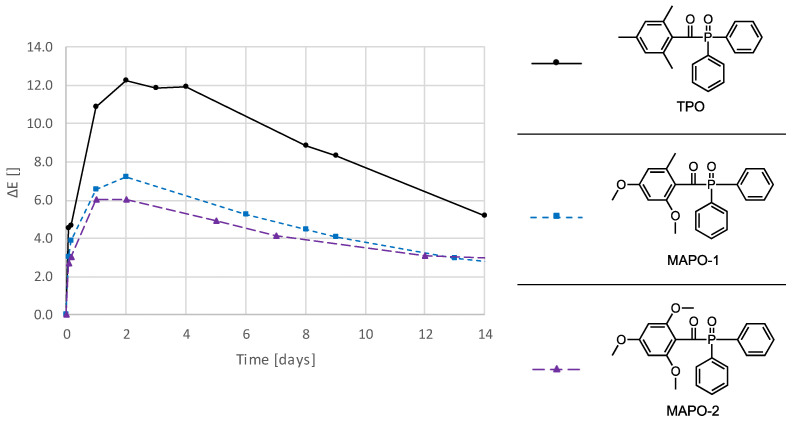
Color changes, ΔE, of samples consisting of UDMA (stabilized with 100 ppm MeHQ) and 1 mol% of the different photoinitiators TPO, MAPO-1, and MAPO-2. The samples were stored at 50 °C immersed in water.

The preliminary tests (0) show that the use of different photoinitiators causes major changes in the resulting absorption spectrum. Therefore, photoinitiators were sought that behave similar to TPO but cause less discoloration. In contrast to TPO, the synthesized photoinitiators MAPO-1 and MAPO-2 have a more sterically hindered benzoyl chromophore. It is assumed that this steric requirement makes the benzoyl chromophore less accessible for subsequent reactions, thus suppressing the formation of colored secondary products. For the comparison with TPO, samples with an equimolar photoinitiator content of 1 mol% in UDMA (stabilized with 100 ppm MeHQ) were prepared and evaluated with CIELAB parameters. Figure 5 shows the color change (ΔE) and the results for ΔL, Δa, and Δb, and L, a, and b are shown in Figure S8 and Figure S9. It can be seen that MAPO-1 and MAPO-2 had a distinctly higher color stability compared to TPO, with MAPO-2 performing slightly better than MAPO-1.

### 3.4. Addition of Stabilizers

In the preliminary tests (0), it was shown that the use of purified UDMA results in stronger discoloration. One possible explanation for this behavior could be the absence of the inhibitor MeHQ. To investigate this parameter, a thin-film sample consisting of TPO, UDMA (stabilized with 100 ppm MeHQ), and an additional 300 ppm of the inhibitor MeHQ was firstly prepared and examined with regard to the discoloration behavior (Figure S10). Hardly any differences were observed in the initial discoloration behavior, but from day 10 onwards, it was observed that the sample with additional MeHQ was already decolorizing, while the absorbance of the standard sample was still increasing. The stabilizer could trap residual free benzoyl radicals, preventing them from forming colored secondary products and, thus, reducing the maximum discoloration.

**Figure 6:**
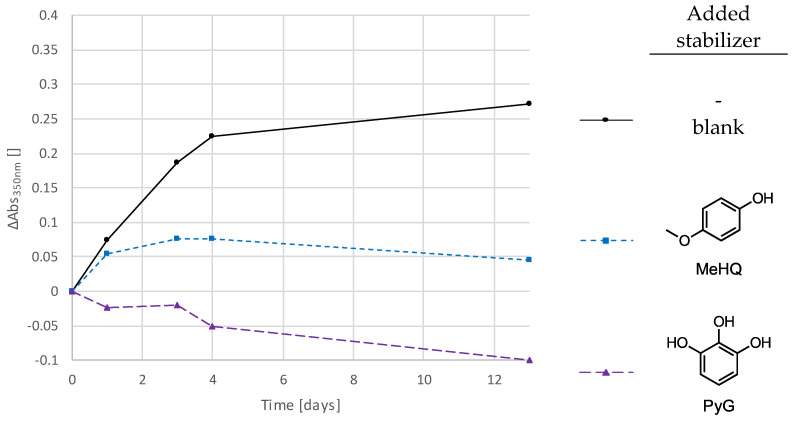
Temporal changes in absorbance at 350 nm of the standard thin-film samples to which 1000 ppm of different stabilizers were added.

Next, a comprehensive study was conducted with different phenolic stabilizers to determine if some are more effective than others in suppressing discoloration. In addition to MeHQ and Pyrogallol (PyG), which are described in more detail here, 3,5-di-*tert*-4-butylhydroxytoluol (BHT), *tert*-butylhydroquinone (TBHQ), (±)-α-tocopherol (TP), pentaerythritol tetrakis[3-(3,5-di-*tert*-butyl-4-hydroxyphenyl)propionate] (PTP), tris(4-*tert*-butyl-3-hydroxy-2,6-dimethylbenzyl)isocyanurate (TIC), and propyl gallate (PrG) were investigated. For this purpose, standard formulations with additional 1000 ppm of the respective stabilizer were prepared and evaluated. Figure S11 shows the absorption spectra of all tested compounds, and Figure 6 shows the corresponding absorbance changes at 350 nm of the most effective ones.

Figure S11 and Figure 6 show that PyG outperforms all other stabilizers and that emerging absorbances can be completely suppressed with it. MeHQ and PrG (Figure S11g–h) also appear to be suitable compounds to reduce color changes. TP, TIC, PTP, BHT, and TBHQ, which, in some cases, improve the discoloration behavior at 350 nm and do not, as observed so far, generate absorption bands at higher wavelengths (Figure S11b–f). These could be due to colored substances that are formed by the stabilizer during the stabilization mechanism, as described in the literature [45,46].

**Figure 7:**
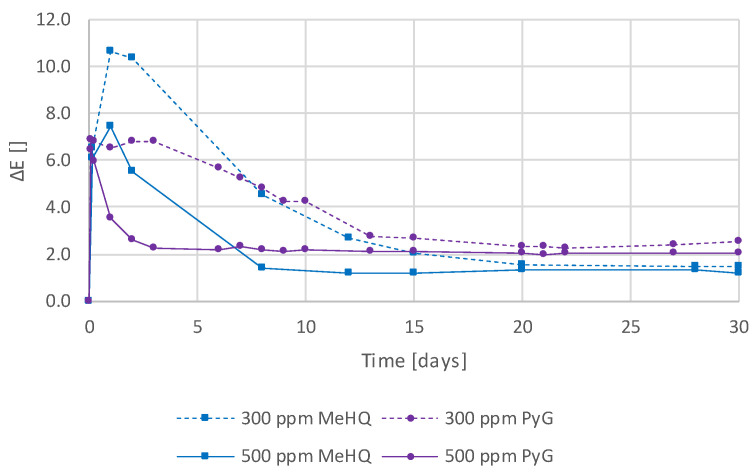
Color changes, ΔE, of the samples consisting of UDMA (without stabilizer), TPO (1 wt% ≙ 1.35 mol%), and 300/500 ppm MeHQ/PyG, stored at 50 °C and immersed in water.

Figure 7 shows the color change (ΔE), and Figure S12 and Figure S13 present the results for the ΔL, Δa, and Δb and the L, a, and b of the CIELAB measurements with the two best-performing stabilizers, MeHQ and PyG, at 300 ppm and 500 ppm, respectively. In addition, the formulations used for these measurements consisted of 1 wt% (≙ 1.35 mol%) of the photoinitiator TPO and nonstabilized UDMA monomer. The use of a nonstabilized monomer ensured that the color stability was based solely on the added stabilizers. The samples were stored at 50 °C and immersed in water. This, again, shows the high color stability due to the addition of these two stabilizers and the superiority of PyG. PyG may more efficiently capture the remaining free benzoyl radicals, which would otherwise lead to discoloration in secondary reactions.

### 3.5. Addition of Hydroperoxide

**Figure 8:**
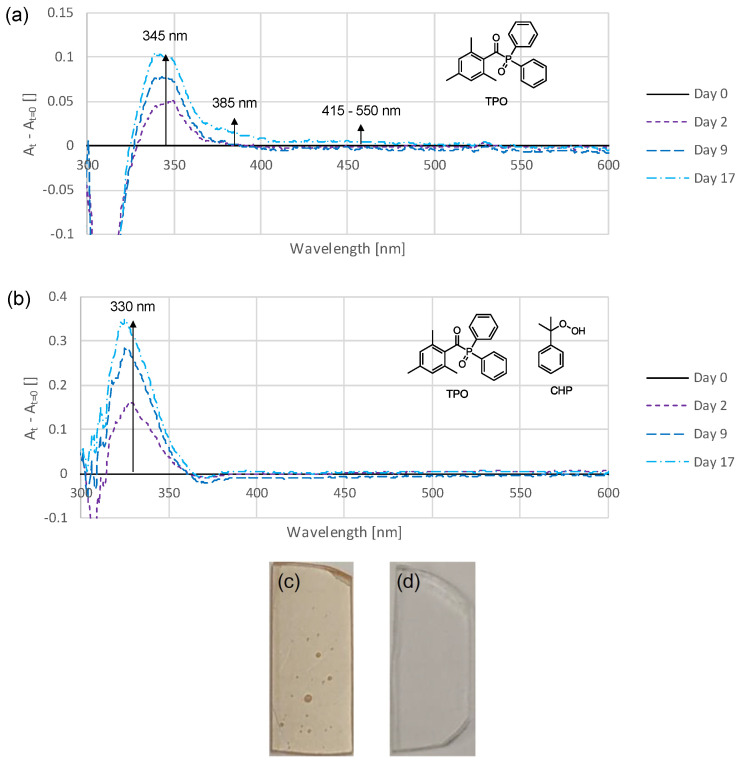
Time-dependent UV-Vis difference spectra of a standard thin-film sample (**a**) and a thin-film sample to which 5 wt% of a CHP solution was added (**b**). Photograph of a standard thin-film sample (**c**) and a thin-film sample to which 5 wt% of a CHP solution was added (**d**) stored under dry conditions for 17 days at 37 °C and for 15 min at 110 °C.

In the preliminary tests (0), it was shown that the samples stored in an oxygen atmosphere discolored significantly less than those stored in an argon atmosphere (Figure 2). The aim of this experiment was to examine whether the addition of hydroperoxides can produce similar effects and perhaps oxidize precursors that could otherwise lead to discoloration. To investigate this, 5 wt% of a cumene hydroperoxide (CHP) solution was added to a standard formulation. The assessment of the sample with regard to the discoloration behavior was carried out using the UV-Vis method. The resulting spectra are shown in Figure 8b in comparison with a reference sample (Figure 8a).

When adding CHP to the standard formulation and during sample preparation, no differences with the reference were observed. During storage, the reference sample showed the greatest increase in absorbance, at around 345 nm. In contrast, the sample containing CHP showed a clear increase in absorbance at 330 nm over time and, thus, was at lower wavelengths compared with the reference. The weaker absorbances at 385 nm and in the range of 415–550 nm observed for the reference sample completely disappeared in the sample containing CHP. To further emphasize this effect, the samples from day 17 were stored at 110 °C for 15 min and then photographed. The difference is clearly visible, while the sample without CHP is colored deep yellow (Figure 8c), the sample with CHP (Figure 8d) appears completely colorless.

## 4. Discussion

From Figure 1, initial conclusions can already be drawn. The first is that the absorption band caused by TPO, which is indicated by the dotted line (before postcuring), disappeared completely after postcuring. Therefore, it can be excluded that discolorations are caused by the remaining photoinitiator. Secondly, it can be seen that, at around 350 nm, an absorbance band formed, which grew over time and then disappeared again over a longer period of time. Therefore, a colored secondary product is expected to form over time, which then slowly degrades or transforms again.

Next, the influences of the different storage conditions were investigated. Figure 2a indicates that wet storage primarily favored the bleaching of the samples, as the initial increase in absorbance was similar for wet and dry storages. Many chemical reactions can be accelerated by increasing the temperature. In this case, it, too, was observed that a higher storage temperature led to faster and stronger discoloration, but also the subsequent bleaching process accelerated (Figure 2b). When investigating the influence of oxygen on the discoloration behavior, oxygen favored the bleaching process, while in the absence of oxygen the observed discoloration was strong and persisted (Figure 2c). Therefore, it is assumed that the bleaching process is based on an oxidation reaction.

The fact that the nature of the monomer matrix influences the discoloration has already been demonstrated in another work [28]. In this work, only D3MA was used for comparison. In the rather hydrophobic D3MA-based thin-film sample, the resulting absorbance was much lower and short-term than in the more polar UDMA based ones. However, it is noteworthy that the resulting absorbance was similar in terms of shift and shape. It seems as if the same discolorations are formed, differing only in intensity. As previously shown, oxygen is a decisive factor for the bleaching behavior. It is possible that the apolar D3MA matrix favors oxygen diffusion in such a way that this causes rapid bleaching.

Next, the purity of the monomer was examined to rule out the possibility that impurities were responsible for the discolorations. For this purpose, UDMA was purified by column chromatography and analyzed with regard to its discoloration behavior. The result of this experiment was initially surprising, because, contrary to the expectation that an impurity might be responsible for the discoloration, pure UDMA discolored more strongly than conventional UDMA (stabilized with 100 ppm MeHQ) (Figure S5). The reason for this was found in the course of the work to be the absence of the stabilizer MeHQ, therefore possible other impurities were not considered in more detail.

Since the discoloration behavior differs quantitatively but not qualitatively in different monomers, the influence of the photoinitiator was investigated. Experiments with thin-films samples containing different concentrations of TPO showed that the strength of the resulting absorption band increased with higher content, but not in a linear relationship, and abruptly with higher concentration (Figure S6). Therefore, it appears that the photoinitiator, or its secondary products are indeed the fundamental cause of the time-dependent discoloration. However, Figure 1 demonstrates that remaining uncleaved photoinitiator is not the source of discoloration, since the characteristic absorption band of TPO vanished entirely after postcuring. Therefore, photolysis, recombination, or other secondary products resulting from the photoinitiator appear to be responsible for the discoloration.

In order to gain deeper insight into the effect of photoinitiators, investigations were carried out in which three photoinitiators that differ fundamentally in terms of their structures, absorptions and the radicals formed were considered. For this purpose, the phosphorus-based photoinitiator TPO was compared with the germanium-based Ivocerin^®^, which also finds application in dentistry, and the carbon-based Darocur 1173 (D1173) which is of great importance in industry. The UV-Vis spectra of the corresponding thin-film samples are shown in Figure 4. These diagrams show that the different initiators also cause differences in the shift and the shape of emerging absorbances.

This led to the suspicion that these discolorations are directly related to the benzoyl chromophore of the photoinitiator. Indeed, this assumption could be confirmed by further thin-film samples. For this purpose, photoinitiators with similar benzoyl chromophores were compared with each other (Figure S7). These experiments show that, for initiators with the same benzoyl chromophore, the resulting absorption bands resembled each other. This also demonstrates that the resulting absorbances in the thin-film samples are independent of the absorption spectrum of the photoinitiator used. For example, the absorption spectra of Ivocerin^®^ (Figure S14b) and I2959 (Figure S14c) are completely different, but produce similar absorption bands

**Figure 9:**
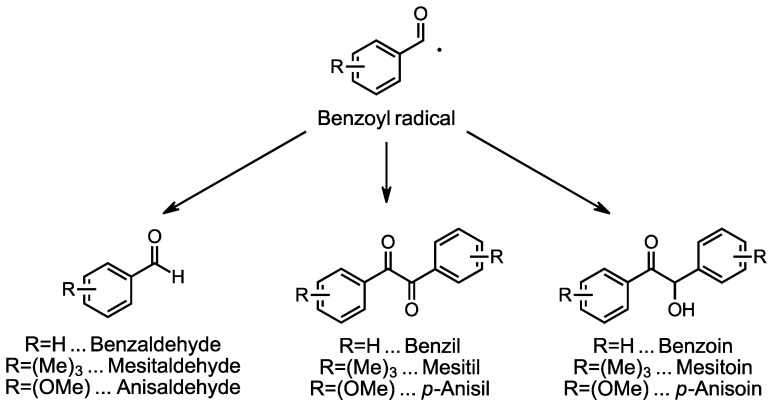
Possible structures that could cause discoloration as a follow-up product of benzoyl radicals.

Initially, the most reasonable explanation for this is that the respective benzaldehyde is formed, or two benzoyl radicals recombine to form the respective benzil or benzoin derivative (Figure 9). The formation of these compounds has already been described in the literature [37,38].

Investigations of this hypothesis by comparison with the UV-Vis spectra of the respective substances could neither clearly confirm nor reject it. For example, in the case of the Ivocerin^®^-based thin film, the absorption spectrum of *p*-anisoin (Figure S16b) appears to fit very well in terms of shape and shift, but the extinction of this substance is very low, so it seems unlikely that it would cause strong discoloration. For D1173, on the other hand, only the spectrum of benzil (Figure S17c) would fit, but here only with regard to the shift and not with regard to the shape. The TPO-based thin film could only be compared with mesitaldehyde (Figure S15) and an absorption spectrum of mesitil from the literature [47]. For mesitoin, no appropriate spectrum was available. In this case, mesitaldehyde seems to be the best match for the observed absorption band in the thin film.

However, none of these substances can explain the weak absorptions at higher wavelengths which were observed with all initiators. Since the time-dependent discoloration behavior of the different initiators matches, it also seems unlikely that different basic substances are responsible for the discoloration. It is assumed that a substance is formed from the benzoyl part of the photoinitiator over a few days, which causes the main absorbance. Either this substance also absorbs light at higher wavelengths, or a follow-up product is formed from it that causes the long-wavelength absorptions.

### 4.1. Photoinitiators for Improved Color Stability

On the one hand, a relationship between discoloration and the benzoyl chromophore of the photoinitiators used could be observed and, on the other hand, secondary products of persistent radicals seem to be responsible for this discoloration. It was therefore considered to modify the benzoyl moiety of the photoinitiator so that the radicals formed cannot undergo further reactions. One possible strategy for this could be to introduce sterically demanding groups and, thus, suppress these follow-up reactions, as the formed radical should be less accessible.

Since the chromophore of the photoinitiator and thus also the resulting absorbance changes compared to TPO, it is difficult to assess the discoloration using the UV-Vis method, which is why the CIELAB color space was used. As photoinitiators with sterically demanding groups, the substances MAPO-1 and MAPO-2 were selected, these are known from literature from the work of Dietlin et al. [43]. As expected, the introduction of sterically demanding groups on the benzoyl moiety had a positive effect on the discoloration behavior (Figure 5). Most likely the methoxy group(s) in ortho position suppresses further reaction of the benzoyl radical to colored compounds. Therefore, the initiator MAPO-2 with two methoxy groups in ortho position shows even a little higher color stability than MAPO-1 with only one *o*-methoxy group.

### 4.2. Addition of Stabilizers for Improved Color Stability

As previously described and shown in Figure S5, it was observed that the purification of the monomer UDMA led to a stronger discoloration. One possible explanation for this could be the absence of the stabilizer MeHQ. To prove this, a formulation with additional MeHQ was produced, whereby the discoloration was significantly reduced (Figure S10).

Subsequently, a wide variety of different stabilizers have been tested (Figure 6 and Figure S11). Differences were found in terms of effectiveness in preventing discoloration, but no clear correlation with the chemical structure could be established. It has been shown that PyG, in particular, but also MeHQ and PrG, is excellent for reducing discoloration. For some stabilizers, namely TP, TIC, PTP, BHT, and TBHQ (Figure S11 b-f), the formation of new absorbances at higher wavelengths was observed, making them unsuitable for the production of unstained samples. This is most likely due to the stabilization mechanism in which colored by-products are formed, which was described for example for BHT by Pospíšil [45].

With the two best performing stabilizers PyG and MeHQ, further tests were carried out in which nonstabilized UDMA monomer was used. Defined quantities of MeHQ and PyG were added and CIELAB measurements of thin films produced from them were carried out (Figure 7). This shows once again that the highest color stability can be achieved by adding PyG. However, very good values were also obtained with MeHQ.

The fact that the presence of stabilizers can reduce discoloration suggests that secondary products of radicals are causing the discoloration. It is possible that the stabilizers trap the remaining radicals of photoinitiators, so that they can no longer form colored follow-up products. In this case, the polymer matrix would affect how easily the radicals can move, or how strongly they are trapped. This in turn would influence how quickly these radicals can react further and thus the time-dependent discoloration.

### 4.3. Addition of Hydroperoxide for Improved Color Stability 

Apart from preventing the development of discoloration, another possibility could be the conversion of discolored species into uncolored ones. As the preliminary tests show, the bleaching of the polymer samples took place quickly in an oxygen atmosphere and almost not at all in the absence of oxygen (Figure 2). It appears that the resulting colored species can be oxidized, making them colorless. It was investigated whether an oxidizing agent could cause the same effect and, thus, suppress discoloration. Figure 8 and Figure 9 show the effect of the addition of the hydroperoxide CHP, which is commonly used in dental materials as oxidizing agent for redox initiator systems. This effect is remarkable, it seems to shift the absorbance, which otherwise occurs at around 345 nm to 330 nm. The weaker absorptions that normally occur at higher wavelengths are, apparently, completely suppressed by the addition of CHP.

This experiment proves that the concept of adding hydroperoxides to prevent discoloration is effective. It appears that CHP immediately oxidizes the resulting discoloration substance and, thus, shifts its absorbance to the nonvisible range. The absence of the longer wavelength absorptions could indicate that the normally observed coloration component can also generate strongly colored secondary products.

After this first experiment, the addition of hydroperoxides seems to effectively prevent discoloration, so further research with other oxidizing agents and lower concentrations would certainly be interesting.

## 5. Conclusions

The time-dependent discoloration behavior of light-cured polymers is influenced by several factors. The storage conditions (temperature, oxygen content, and dry/wet storage) were shown to play a significant role. Moreover, the composition of the formulation is also paramount, as the coloration is strongly influenced by the type of monomer and the photoinitiator used. Stronger discoloration was observed with higher photoinitiator content. Furthermore, methods have been found to reduce or completely suppress such discoloration. One possibility is the addition of selected stabilizers (MEHQ and PyG were the most efficient). Another way to reduce this discoloration is through the use of photoinitiators, which exhibit a large steric hindrance on the benzoyl chromophore. The discoloration could even be completely prevented upon addition of the oxidizing agent CHP. Using such technologies, the development of color-stable dental 3D-printing materials is possible.

## Data Availability

Data are contained within the article.

## Supplementary Materials

The following supporting information can be downloaded at: https://www.mdpi.com/article/10.3390/polym16162323/s1, Figure S1: Time-dependent absorption spectra of TPO-based thin-film samples stored under dry (a) and wet (b) conditions; Figure S2: Time-dependent absorption spectra of TPO-based thin-film samples stored at 37 °C (a), 60 °C (b), and 90 °C (c); Figure S3: Time-dependent absorption spectra of TPO-based thin-film samples in presence (dashed lines) and absence of oxygen (solid lines) stored at 37 °C (a), 60 °C (b), and 110 °C (c); Figure S4: Time-dependent absorption spectra of TPO-containing thin-film samples based on UDMA (stabilized with 100 ppm MeHQ) (a) and D3MA (stabilized with 20 ppm BHT) (b). The change in absorbance at 350 nm of these samples over time is shown in (c); Figure S5: Time-dependent absorption spectra of TPO-containing thin-film samples based on conventional UDMA (stabilized with 100 ppm MeHQ) (a) and purified UDMA (b). The change in absorbance at 350 nm of these samples over time is shown in (c); Figure S6: Time-dependent absorption spectra of thin-film samples based containing 0.4 wt% ≙ 0.54 mol% (a), 0.8 wt% ≙ 1.1 mol% (b) and 1.2 wt% ≙ 1.62 mol% (c) of the photoinitiator TPO. The change in absorbance at 350 nm of these samples over time is shown in (c); Figure S7: Time-dependent absorption spectra of thin-film samples based containing different photoinitiators with comparable benzoyl chromophores. TPO (1.1 mol%) (a)/BAPO (1.1 mol%) (b), Ivocerin® (1.1 mol%) (c)/I2959 (2.2 mol%) (d), and D1173 (1.1 mol%) (e)/I184 (1.1 mol%) (f) in comparison with each other; Figure S8: Change in lightness, ΔL, (a), green–red error Δa (b), and blue–yellow error Δb (c) of the samples consisting of UDMA (stabilized with 100 ppm MeHQ) and 1 mol% respective photoinitiator. The thin-film samples were immersed in water and stored at 50 °C; Figure S9: L values (a), a values (b), and b values (c) of the samples consisting of UDMA (stabilized with 100 ppm MeHQ) and 1 mol% respective photoinitiator. The thin-film samples were immersed in water and stored at 50 °C; Figure S10: Time-dependent absorption spectra of thin-film samples of a standard formulation (a) and a formulation to which 300 ppm MeHQ was added (b). The change in absorbance at 350 nm for these samples over time is shown in (c); Figure S11: Time-dependent absorbance spectra of thin-film samples with a standard formulation (a) and with the addition of 1000 ppm of the respective stabilizer (b-i). The change in absorbances at 350 nm of these samples over time is shown in (j); Figure S12: Change in lightness ΔL (a), green-red error Δa (b) and blue-yellow error Δb (c) of samples consisting of UDMA (without stabilizer), TPO (1 wt% ≙ 1.35 mol%) and 300 ppm/500 ppm MeHQ/PyG, stored at 50 °C immersed in water; Figure S13: L values (a), a values (b) and b values (c) of samples consisting of UDMA (without stabilizer), TPO (1 wt% ≙ 1.35 mol%) and 300/500 ppm MeHQ/PyG, stored at 50 °C immersed in water; Figure S14: UV-Vis absorption spectra of the photoinitiators TPO (a), Ivocerin® (b), and I2959 (c) in acetonitrile solution; Figure S15: UV Vis absorption spectrum of mestitaldehyde in acetonitrile solution; Figure S16: UV Vis absorption spectra of anisaldehyde (a), p-anisoin (b) and p-anisil (c) in acetonitrile solution; Figure S17: UV Vis absorption spectra of benzaldehyd (a), benzoin (b) and benzill (c) in acetonitrile solution.
