# Supplementary material for: Color-Stable Formulations for 3D-Photoprintable Dental Materials
Source: Polymers (Basel). 2024 Aug 16;16(16):2323. doi: 10.3390/polym16162323 (PMC11359577; doi:10.3390/polym16162323)
Supplement: Supplementary file 1 [file polymers-16-02323-s001.zip › polymers-3138158-supplementary.pdf]

Supporting Material: Color-Stable Formulations for 3D-Photoprintable Dental Materials

David Bassenheim, Kai Rist, Norbert Moszner, Yohann Catel, Robert Liska, Patrick Knaack

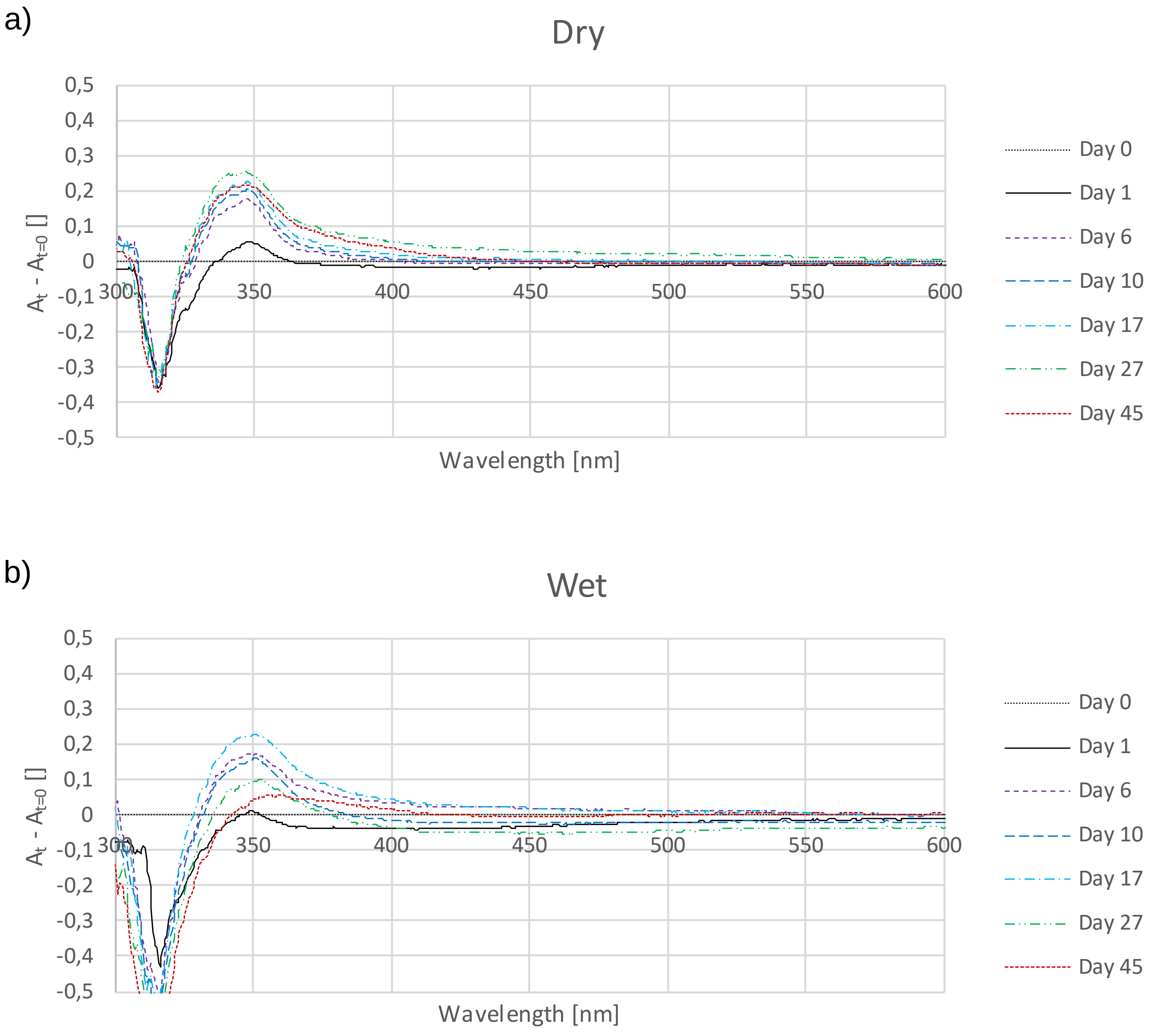

Figure S1: Time-dependent absorption spectra of TPO-based thin-film samples stored under dry (**a**) and wet (**b**) conditions.

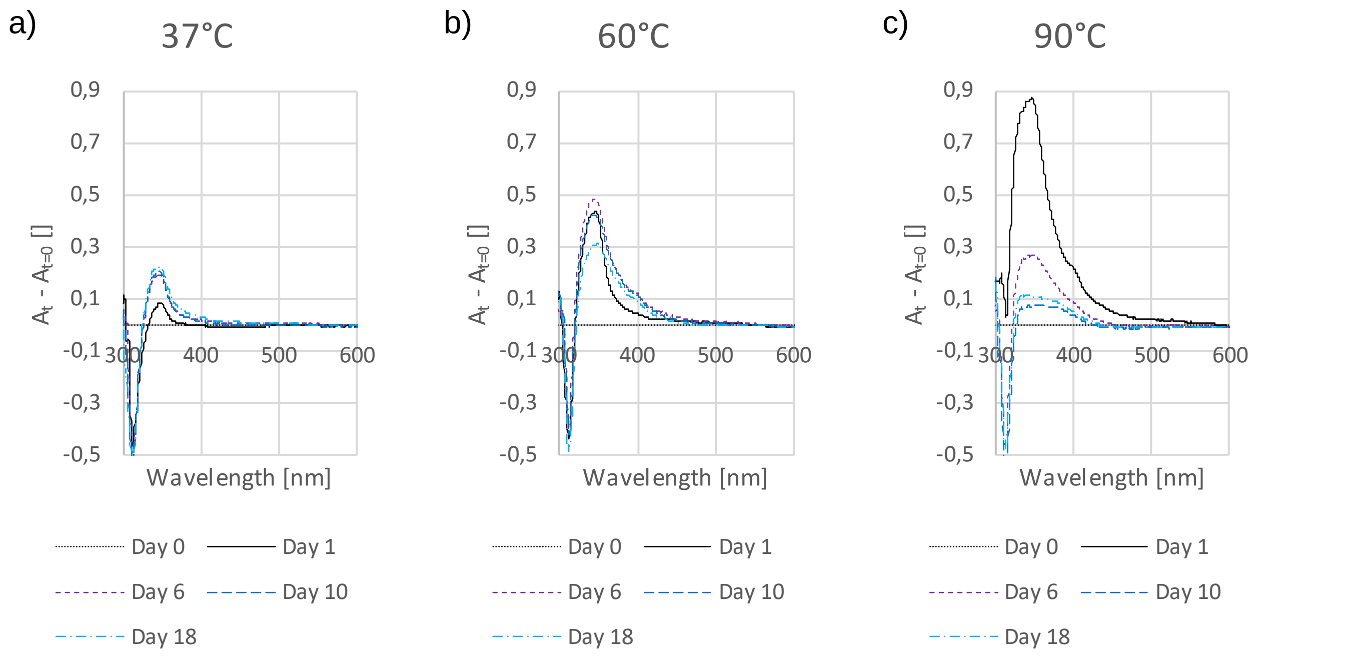

Figure S2: Time-dependent absorption spectra of TPO-based thin-film samples stored at 37 °C (**a**), 60 °C (**b**), and 90 °C (**c**).

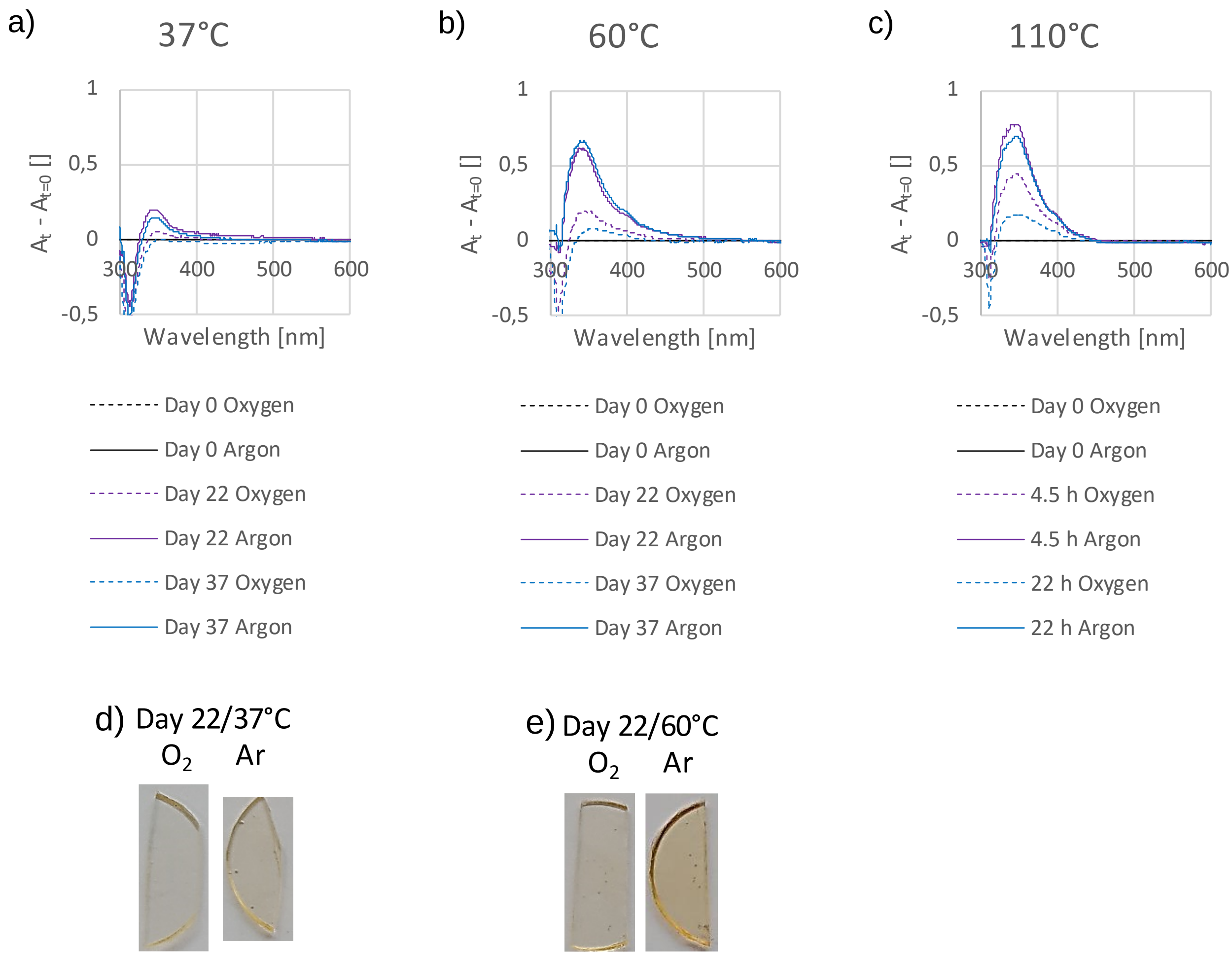

Figure S3: Time-dependent absorption spectra of TPO-based thin-film samples in the presence (dashed lines) and absence of oxygen (solid lines) stored at 37 °C (**a**), 60 °C (**b**), and 110 °C (**c**).

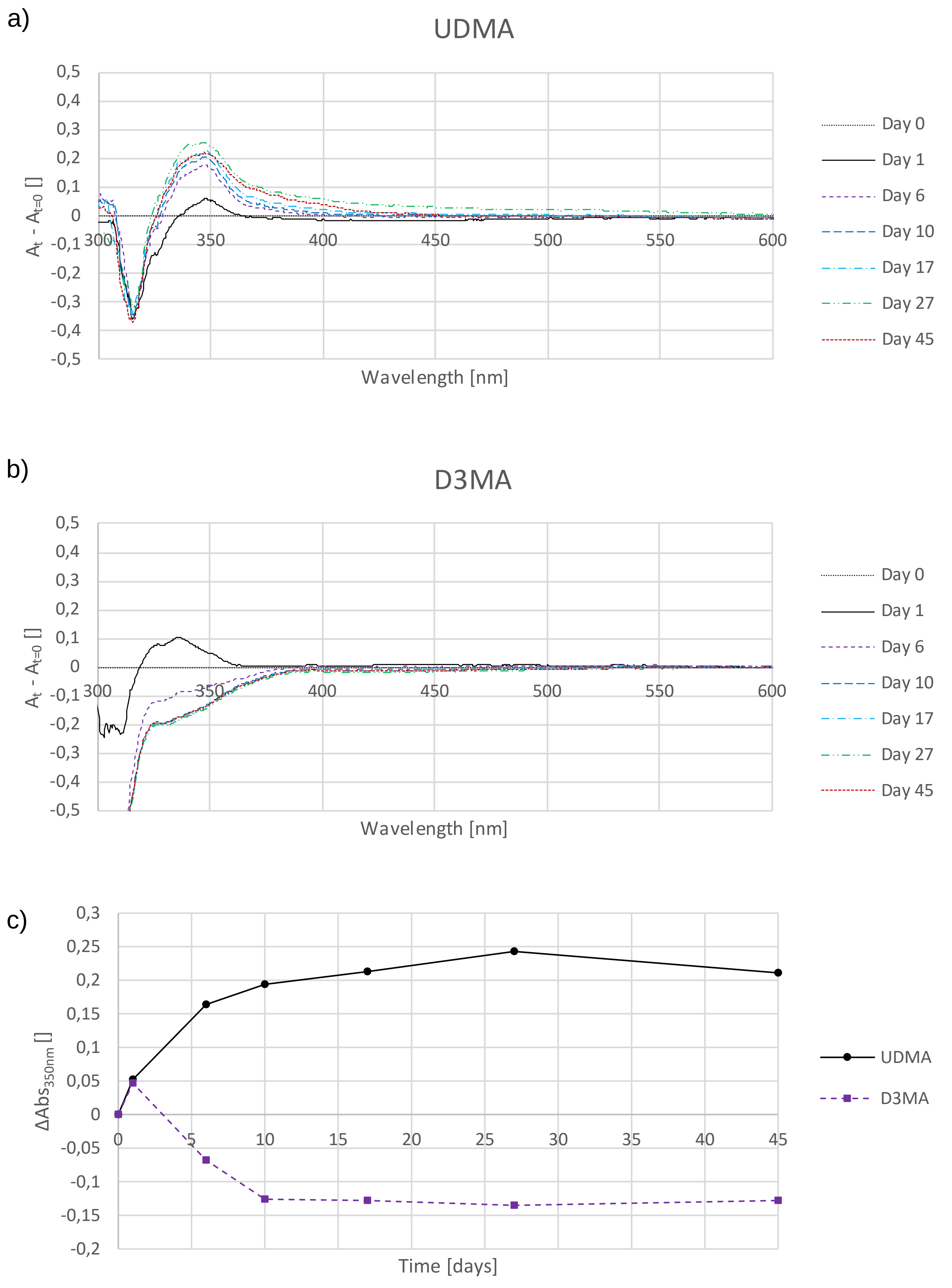

Figure S4: Time-dependent absorption spectra of TPO-containing thin-film samples based on UDMA (stabilized with 100 ppm MeHQ) (**a**) and D3MA (stabilized with 20 ppm BHT) (**b**). The changes in absorbance at 350 nm of these samples over time are shown in (**c**).

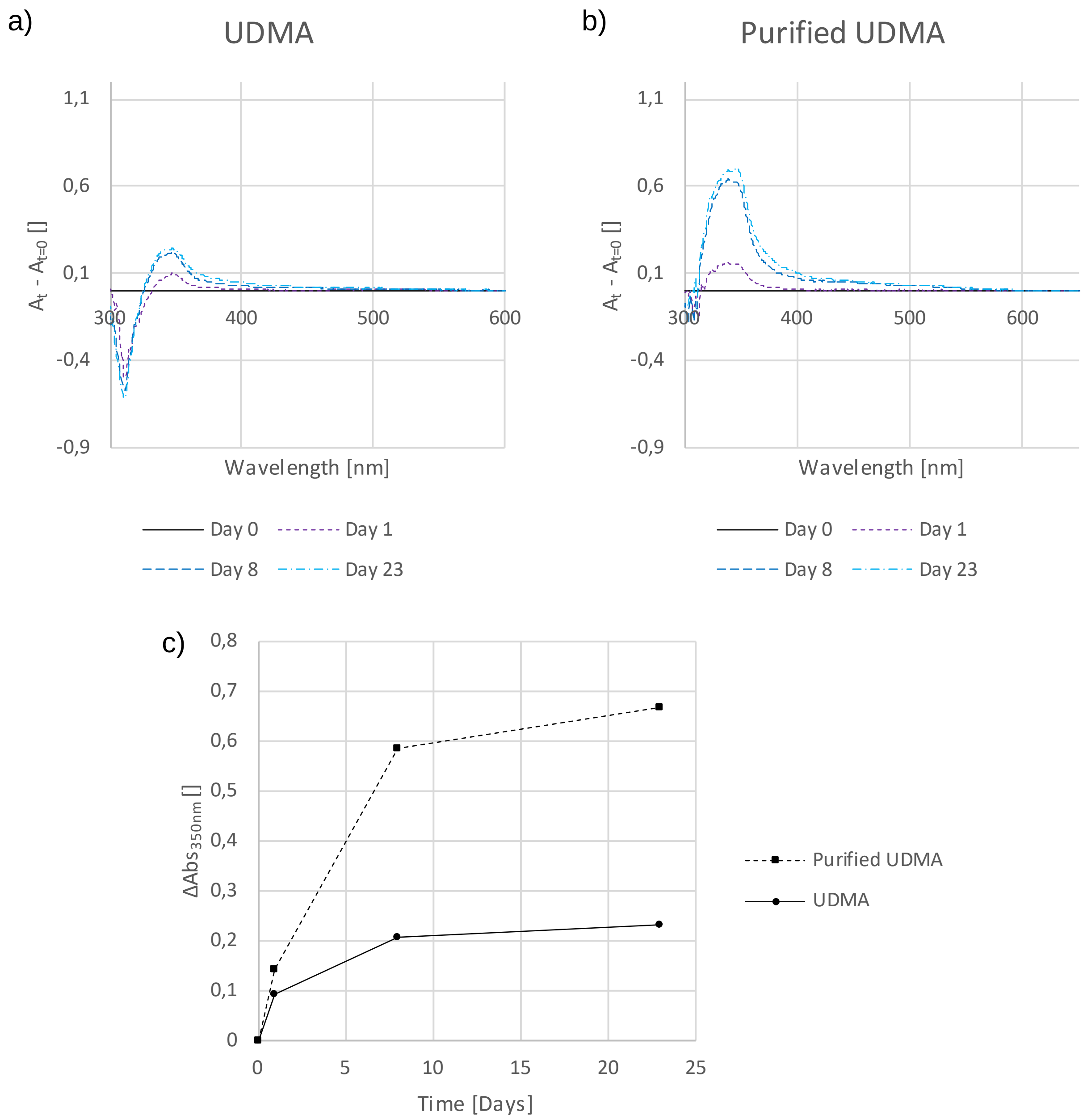

Figure S5: Time-dependent absorption spectra of TPO-containing thin-film samples based on conventional UDMA (stabilized with 100 ppm MeHQ) (**a**) and purified UDMA (**b**). The changes in absorbance at 350 nm of these samples over time are shown in (**c**).

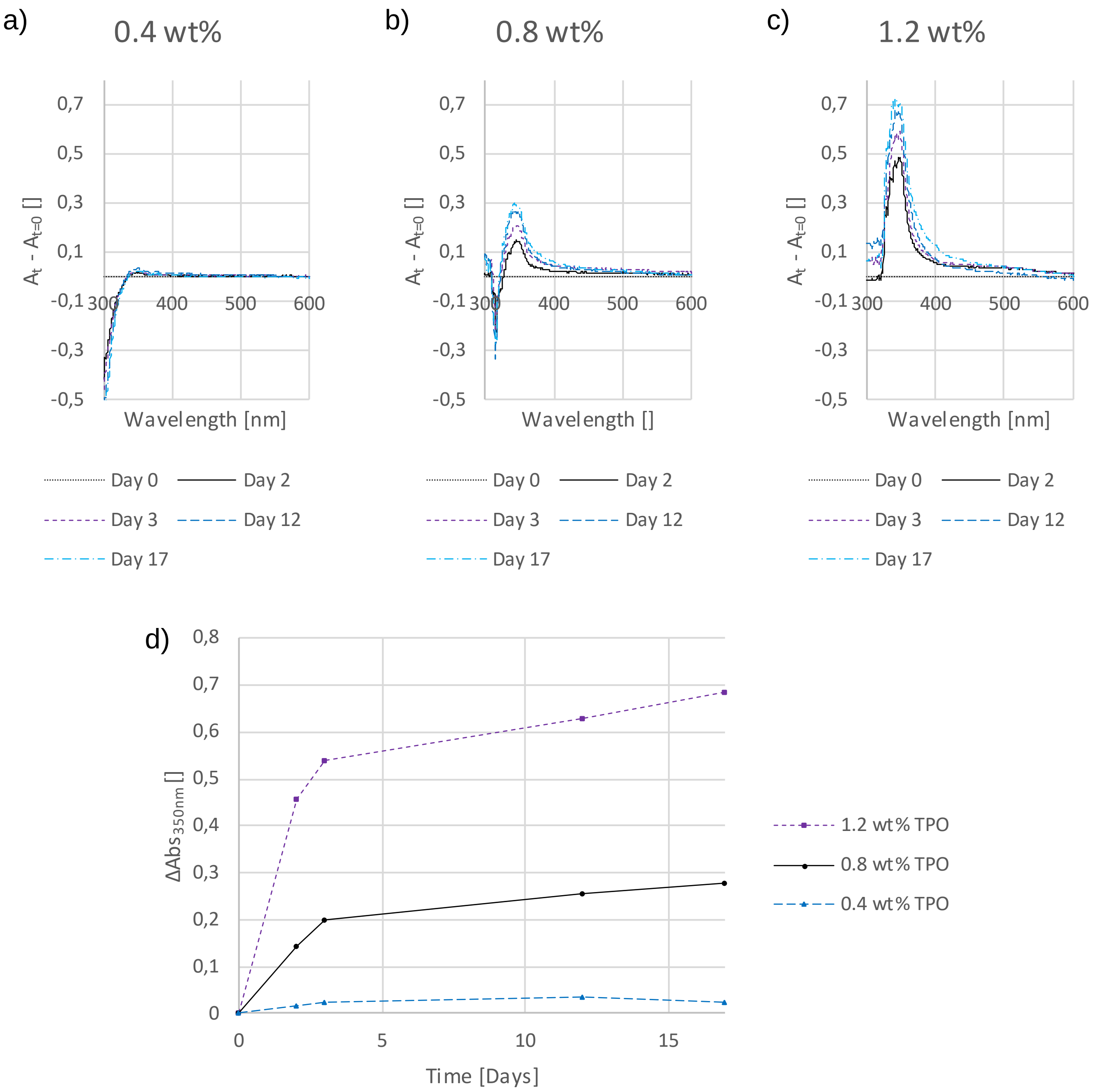

Figure S6: Time-dependent absorption spectra of thin-film samples containing 0.4 wt% ≙ 0.54 mol% (**a**), 0.8 wt% ≙ 1.1 mol% (**b**), and 1.2 wt% ≙ 1.62 mol% (**c**) of the photoinitiator TPO. The changes in absorbance at 350 nm of these samples over time are shown in (**c**).

a)

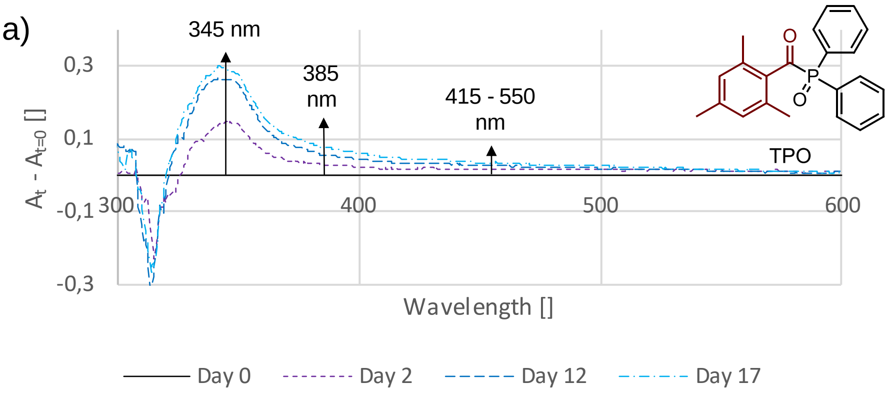

345 nm
385 nm
415 - 550 nm
TPO
0,3
0,1
-0,1
-0,3
300
400
500
600
$A_t - A_{t=0}$ []
Wavelength []
Day 0
Day 2
Day 12
Day 17

b)

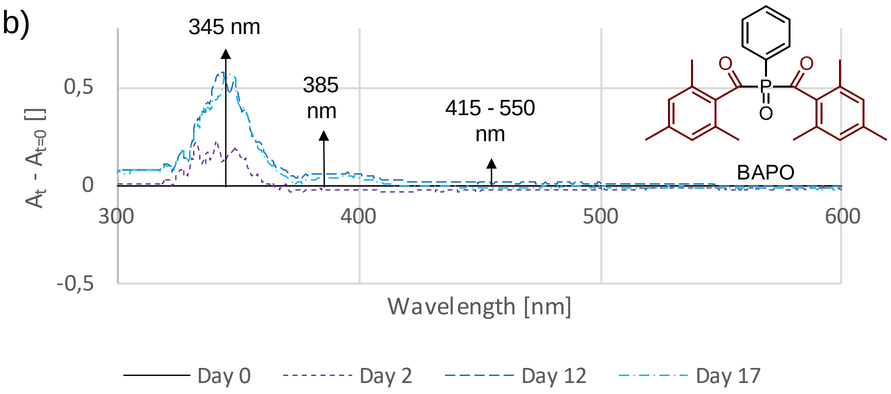

345 nm
385 nm
415 - 550 nm
BAPO
0,5
0
-0,5
300
400
500
600
$A_t - A_{t=0}$ []
Wavelength [nm]
Day 0
Day 2
Day 12
Day 17

c)

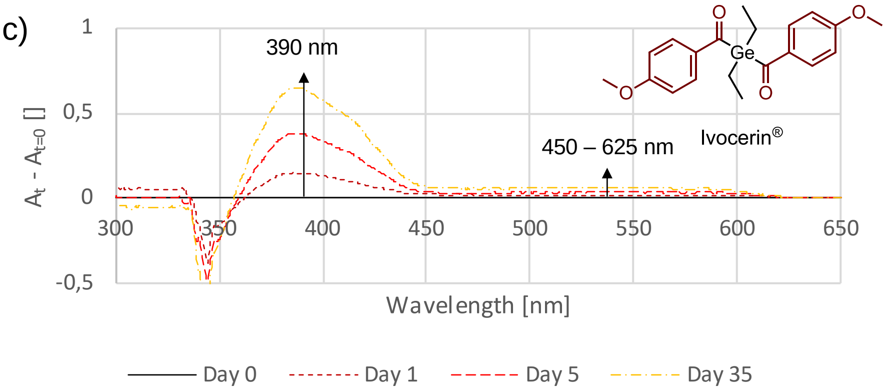

390 nm
450 – 625 nm
Ivocerin®
1
0,5
0
-0,5
300
350
400
450
500
550
600
650
$A_t - A_{t=0}$ []
Wavelength [nm]
Day 0
Day 1
Day 5
Day 35

d)

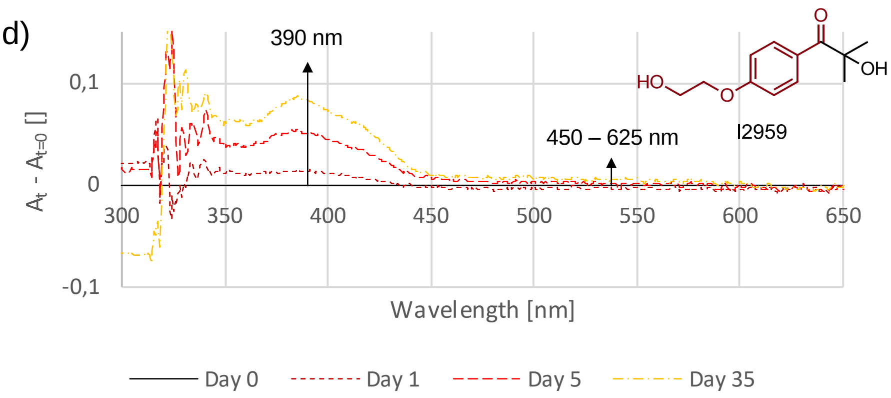

390 nm
450 – 625 nm
I2959
HO
OH
0,1
0
-0,1
300
350
400
450
500
550
600
650
$A_t - A_{t=0}$ []
Wavelength [nm]
Day 0
Day 1
Day 5
Day 35

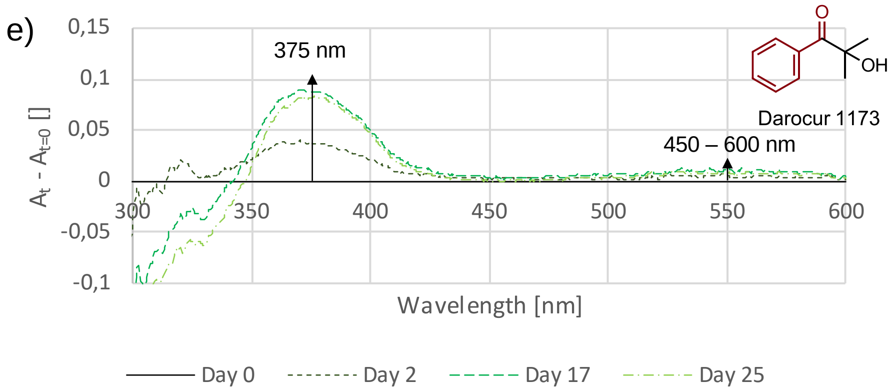

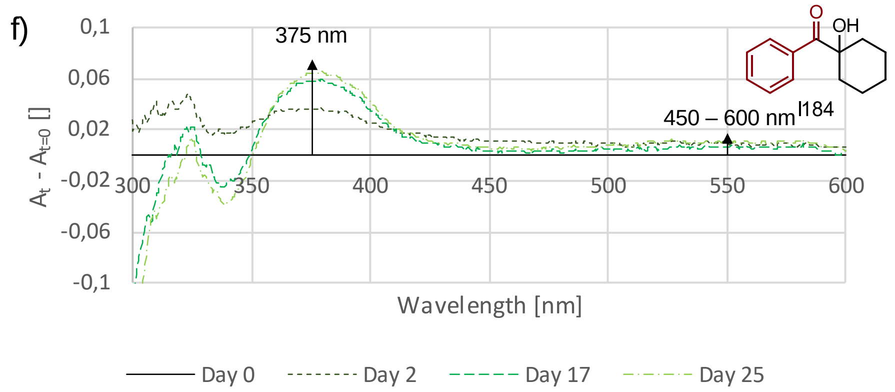

Figure S7: Time-dependent absorption spectra of thin-film samples containing different photoinitiators with comparable benzoyl chromophores: TPO (1.1 mol%) (**a**); / BAPO (1.1 mol%) (**b**); Ivocerin® (1.1 mol%) (**c**); / I2959 (2.2 mol%) (**d**); D1173 (1.1 mol%) (**e**); / I184 (1.1 mol%) (**f**), in comparison with each other.

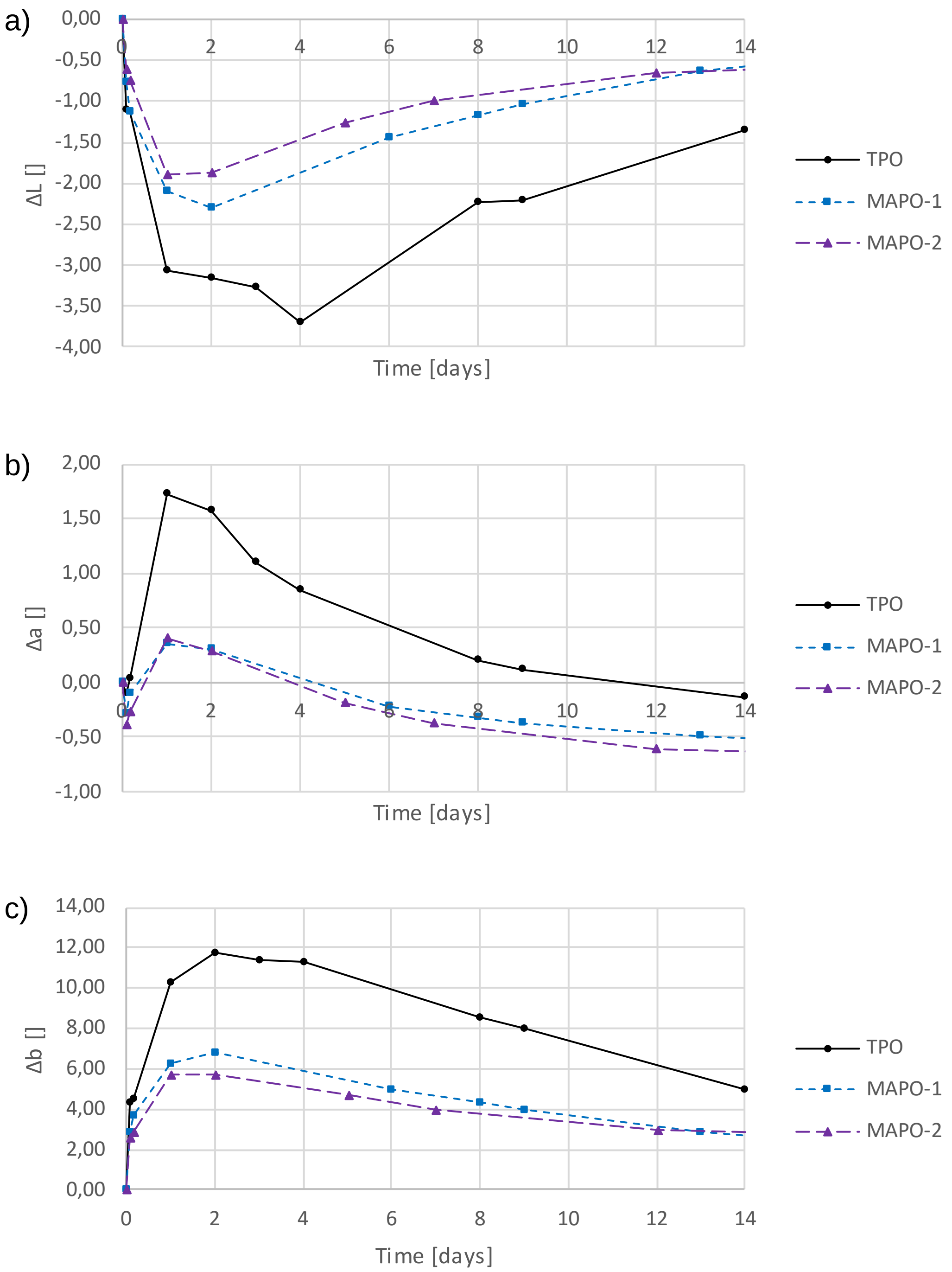

Figure S8: Change of lightness ΔL (**a**), green–red error Δa (**b**), and blue–yellow error Δb (**c**) of samples consisting of UDMA (stabilized with 100 ppm MeHQ) and 1 mol% of the respective photoinitiator. The thin-film samples were immersed in water and stored at 50 °C.

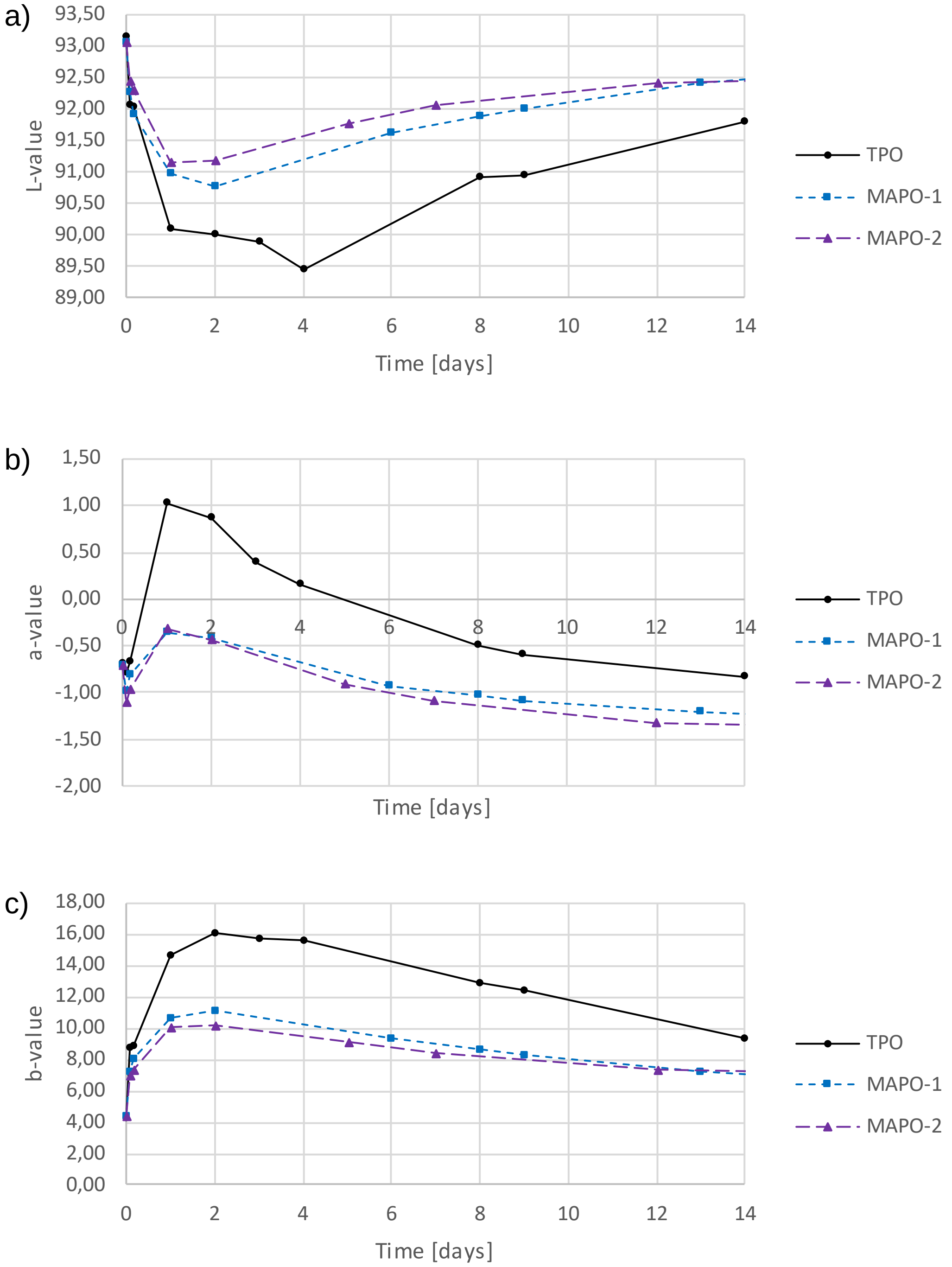

Figure S9: L-values (**a**), a-values (**b**), and b-values (**c**) of samples consisting of UDMA (stabilized with 100 ppm MeHQ) and 1 mol% of the respective photoinitiator. The thin-film samples were immersed in water and stored at 50 °C.

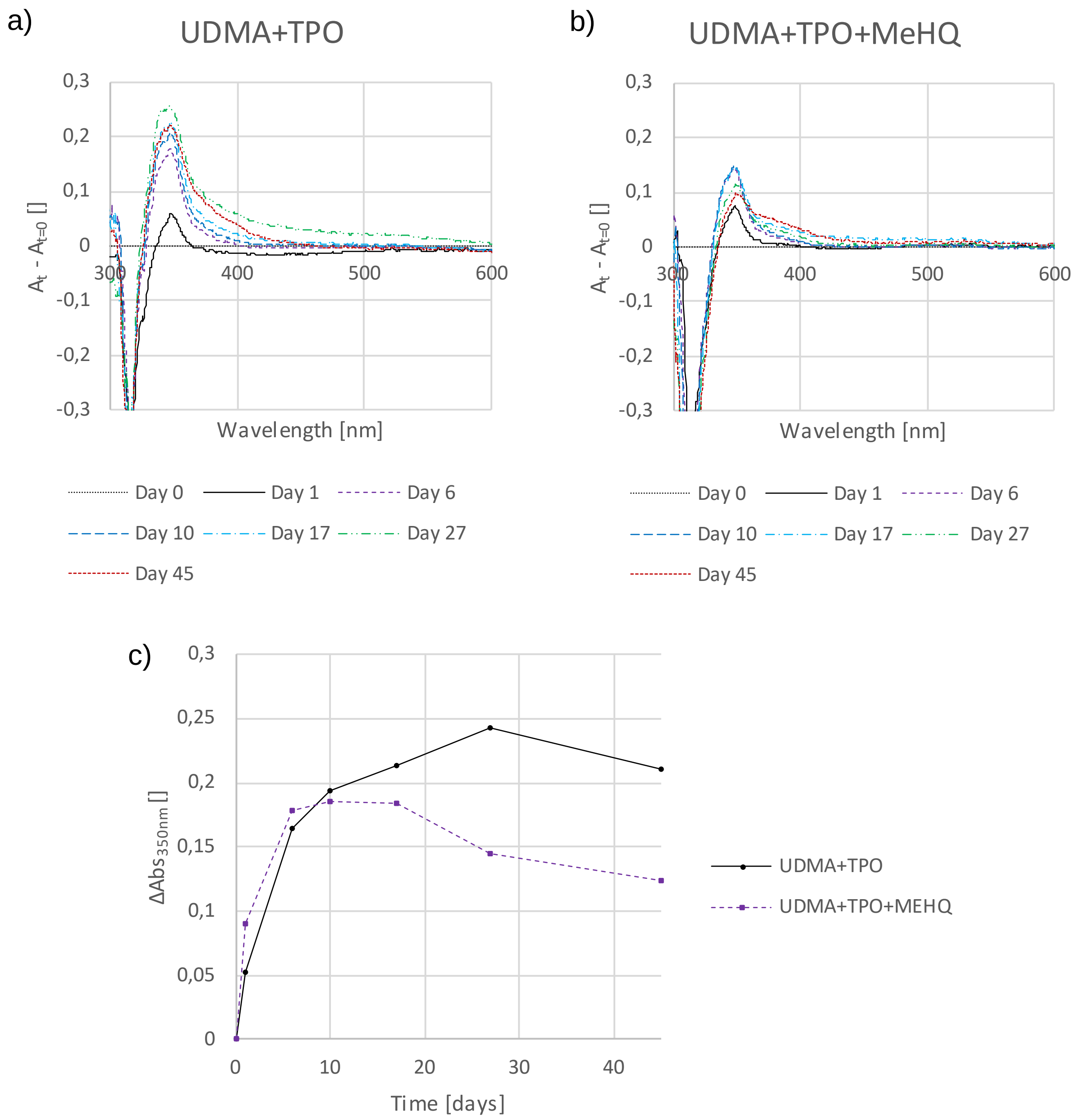

Figure S10: Time-dependent absorption spectra of thin-film samples of a standard formulation (**a**) and a formulation to which 300 ppm MeHQ were added (**b**). The changes in absorbance at 350 nm by these samples over time are shown in (**c**).

a)

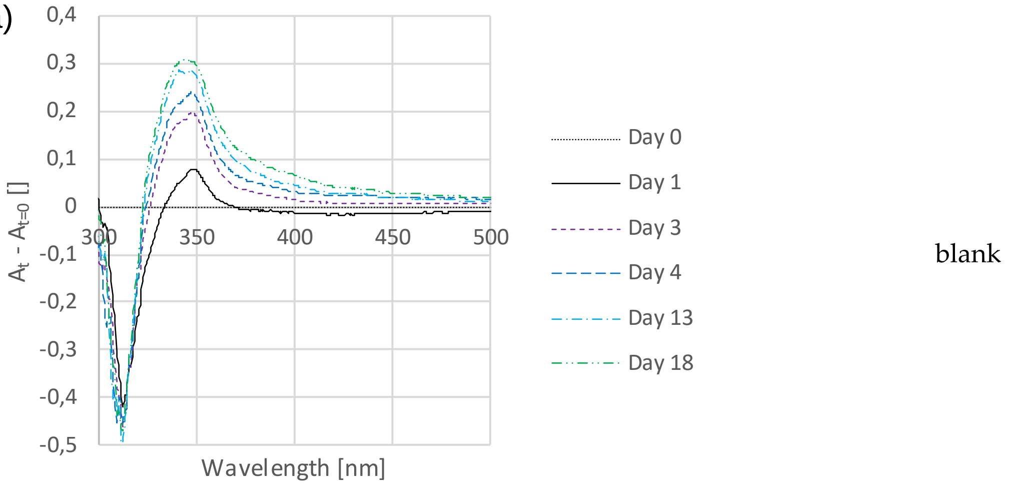

0,4
0,3
0,2
0,1
0
-0,1
-0,2
-0,3
-0,4
-0,5
300
350
400
450
500
A_t - $A_{t=0}$ []
Wavelength [nm]
Day 0
Day 1
Day 3
Day 4
Day 13
Day 18
blank

b)

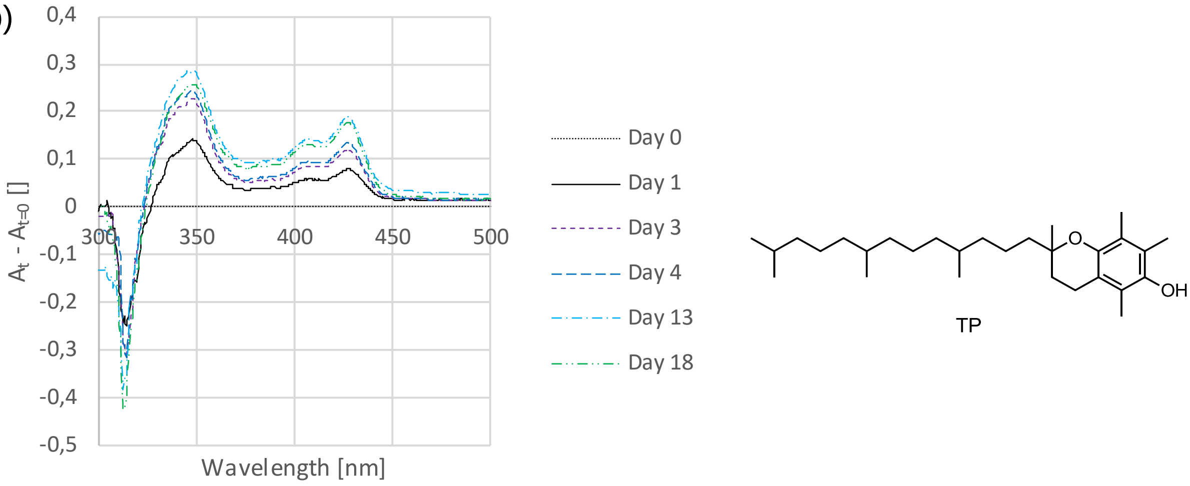

0,4
0,3
0,2
0,1
0
-0,1
-0,2
-0,3
-0,4
-0,5
300
350
400
450
500
A_t - $A_{t=0}$ []
Wavelength [nm]
Day 0
Day 1
Day 3
Day 4
Day 13
Day 18
O
OH
TP

c)

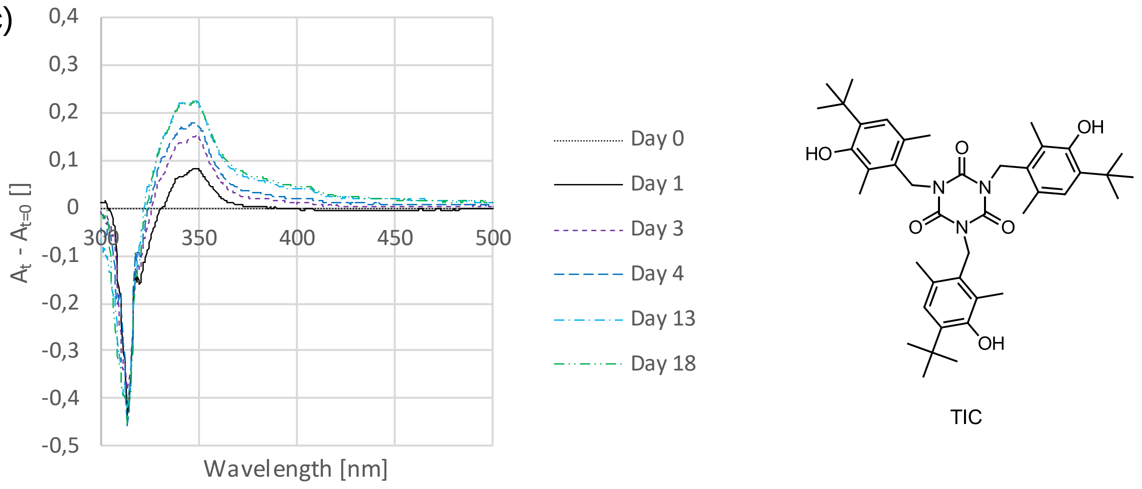

0,4
0,3
0,2
0,1
0
-0,1
-0,2
-0,3
-0,4
-0,5
300
350
400
450
500
A_t - $A_{t=0}$ []
Wavelength [nm]
Day 0
Day 1
Day 3
Day 4
Day 13
Day 18
HO
OH
OH
N
O
TIC

d)

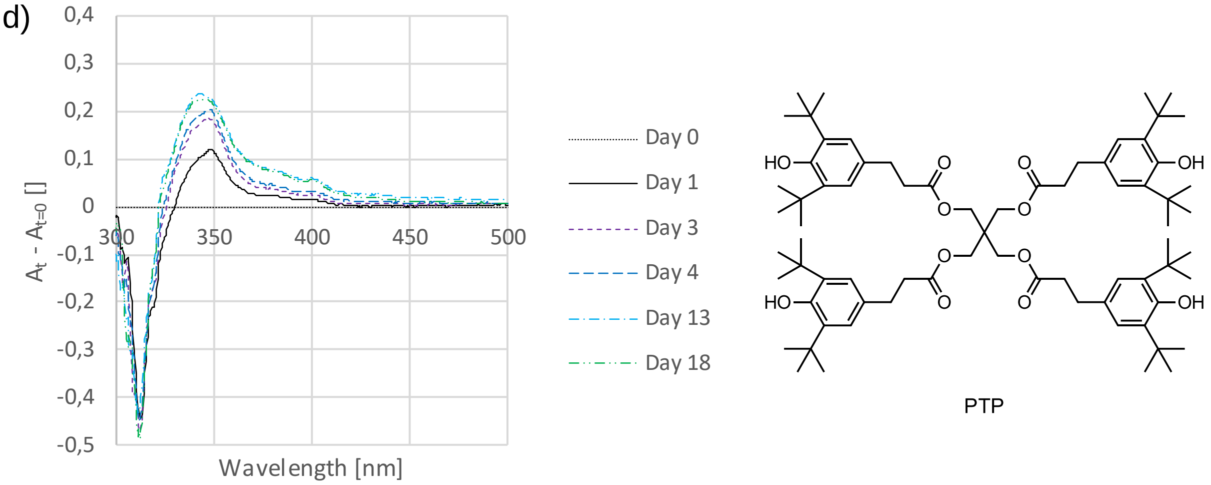

0,4
0,3
0,2
0,1
0
-0,1
-0,2
-0,3
-0,4
-0,5
300
350
400
450
500
At - At=0 []
Wavelength [nm]
Day 0
Day 1
Day 3
Day 4
Day 13
Day 18
HO
OH
O
PTP

e)

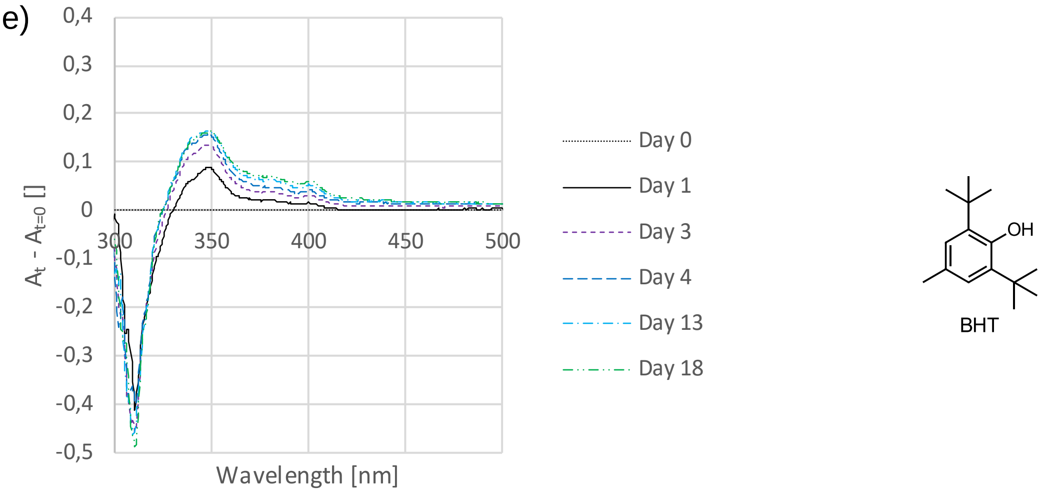

0,4
0,3
0,2
0,1
0
-0,1
-0,2
-0,3
-0,4
-0,5
300
350
400
450
500
At - At=0 []
Wavelength [nm]
Day 0
Day 1
Day 3
Day 4
Day 13
Day 18
OH
BHT

f)

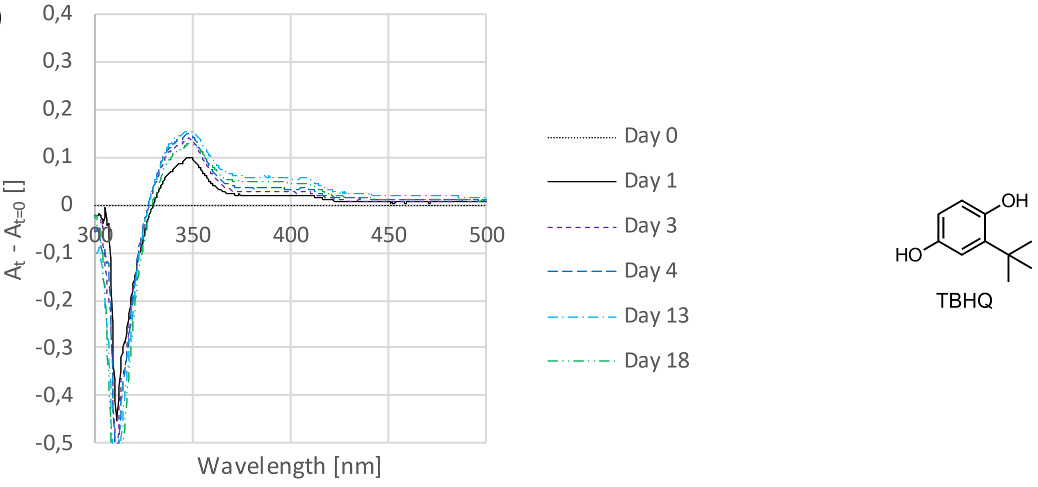

0,4
0,3
0,2
0,1
0
-0,1
-0,2
-0,3
-0,4
-0,5
300
350
400
450
500
At - At=0 []
Wavelength [nm]
Day 0
Day 1
Day 3
Day 4
Day 13
Day 18
OH
HO
TBHQ

g)

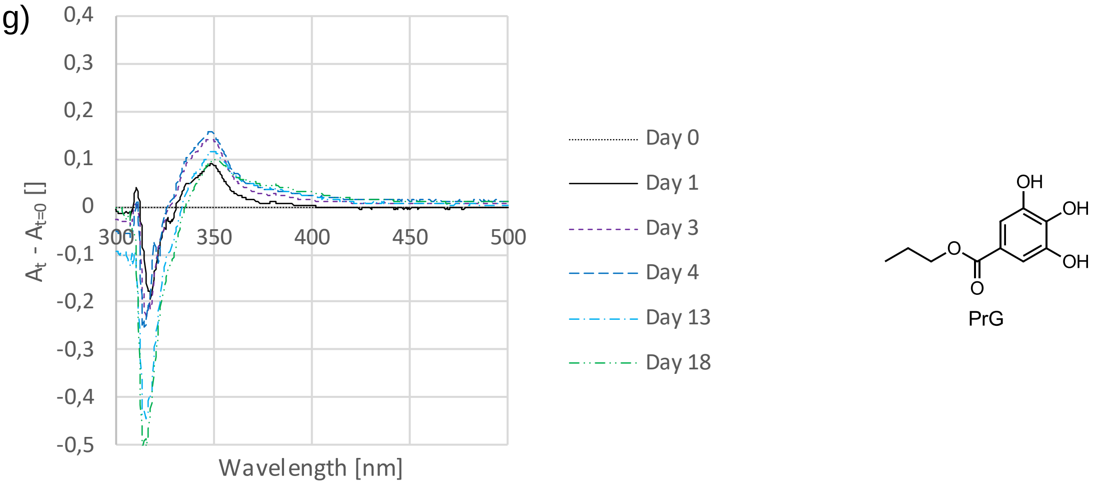

0,4
0,3
0,2
0,1
0
-0,1
-0,2
-0,3
-0,4
-0,5
300
350
400
450
500
A_t - $A_{t=0}$ []
Wavelength [nm]
Day 0
Day 1
Day 3
Day 4
Day 13
Day 18
OH
OH
OH
O
O
PrG

h)

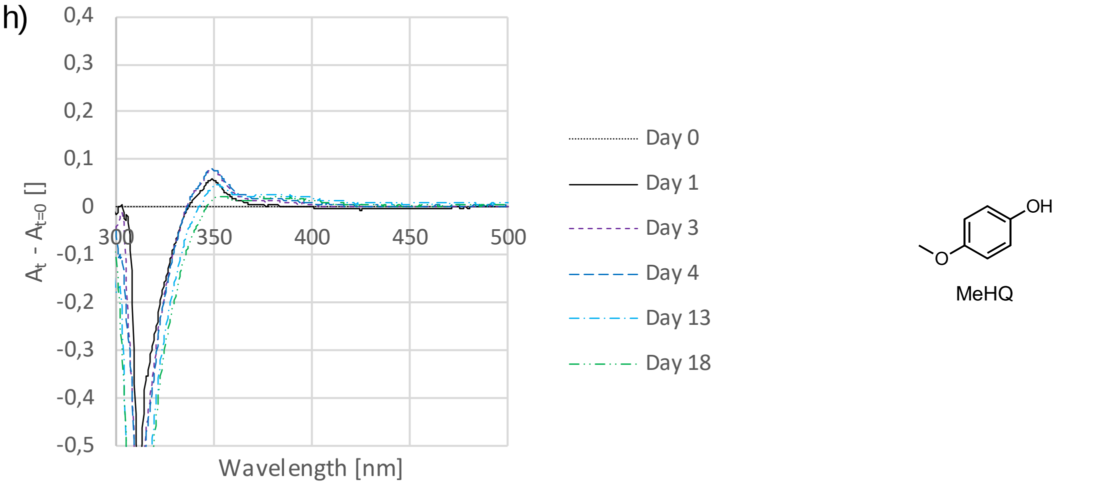

0,4
0,3
0,2
0,1
0
-0,1
-0,2
-0,3
-0,4
-0,5
300
350
400
450
500
A_t - $A_{t=0}$ []
Wavelength [nm]
Day 0
Day 1
Day 3
Day 4
Day 13
Day 18
OH
O
MeHQ

i)

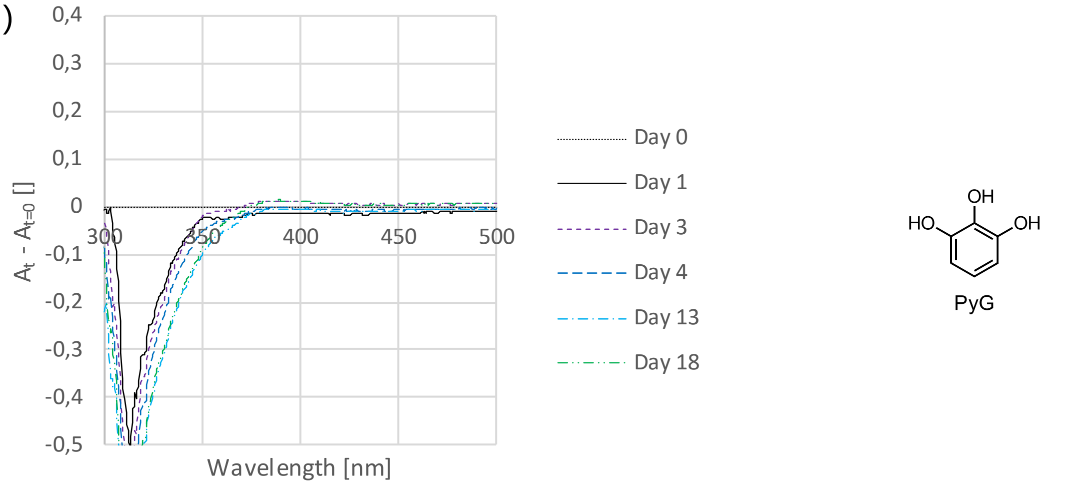

0,4
0,3
0,2
0,1
0
-0,1
-0,2
-0,3
-0,4
-0,5
300
350
400
450
500
A_t - $A_{t=0}$ []
Wavelength [nm]
Day 0
Day 1
Day 3
Day 4
Day 13
Day 18
OH
HO
OH
PyG

j)

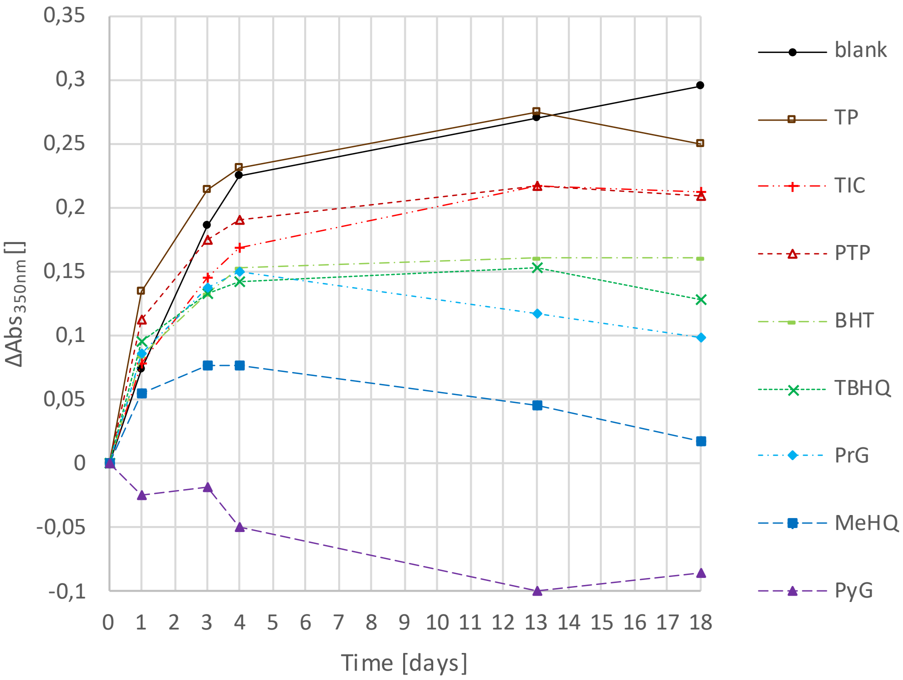

Figure S11: Time-dependent absorbance spectra of thin-film samples with a standard formulation (**a**) and with the addition of 1000 ppm of the respective stabilizer (**b–i**). The changes in absorbance at 350 nm by these samples over time are shown in (**j**).

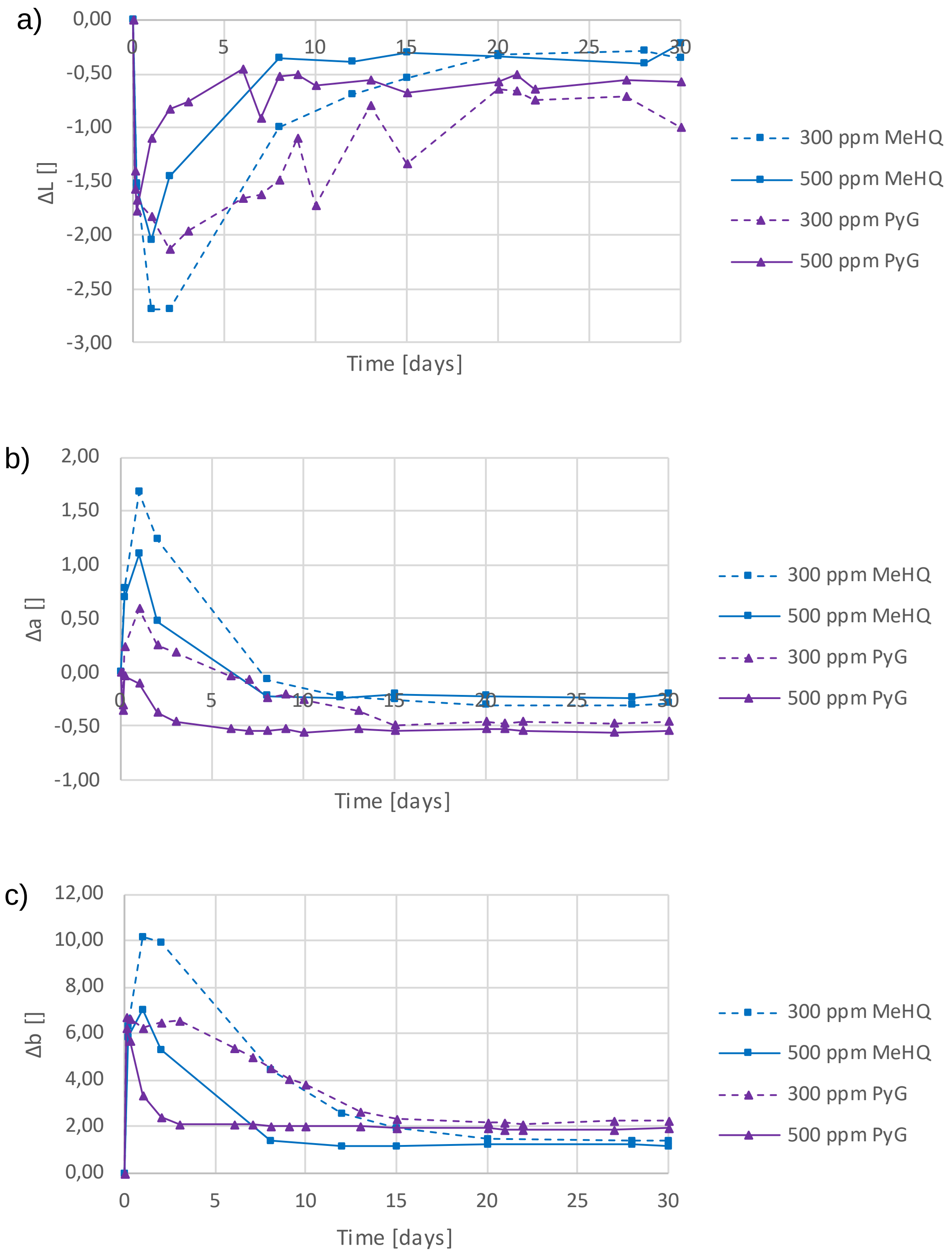

Figure S12: Change of lightness ΔL (**a**), green–red error Δa (**b**), and blue–yellow error Δb (**c**) of samples consisting of UDMA (without stabilizer), TPO (1 wt% ≙ 1.35 mol%), and 300 ppm/500 ppm MeHQ/PyG, stored at 50 °C immersed in water.

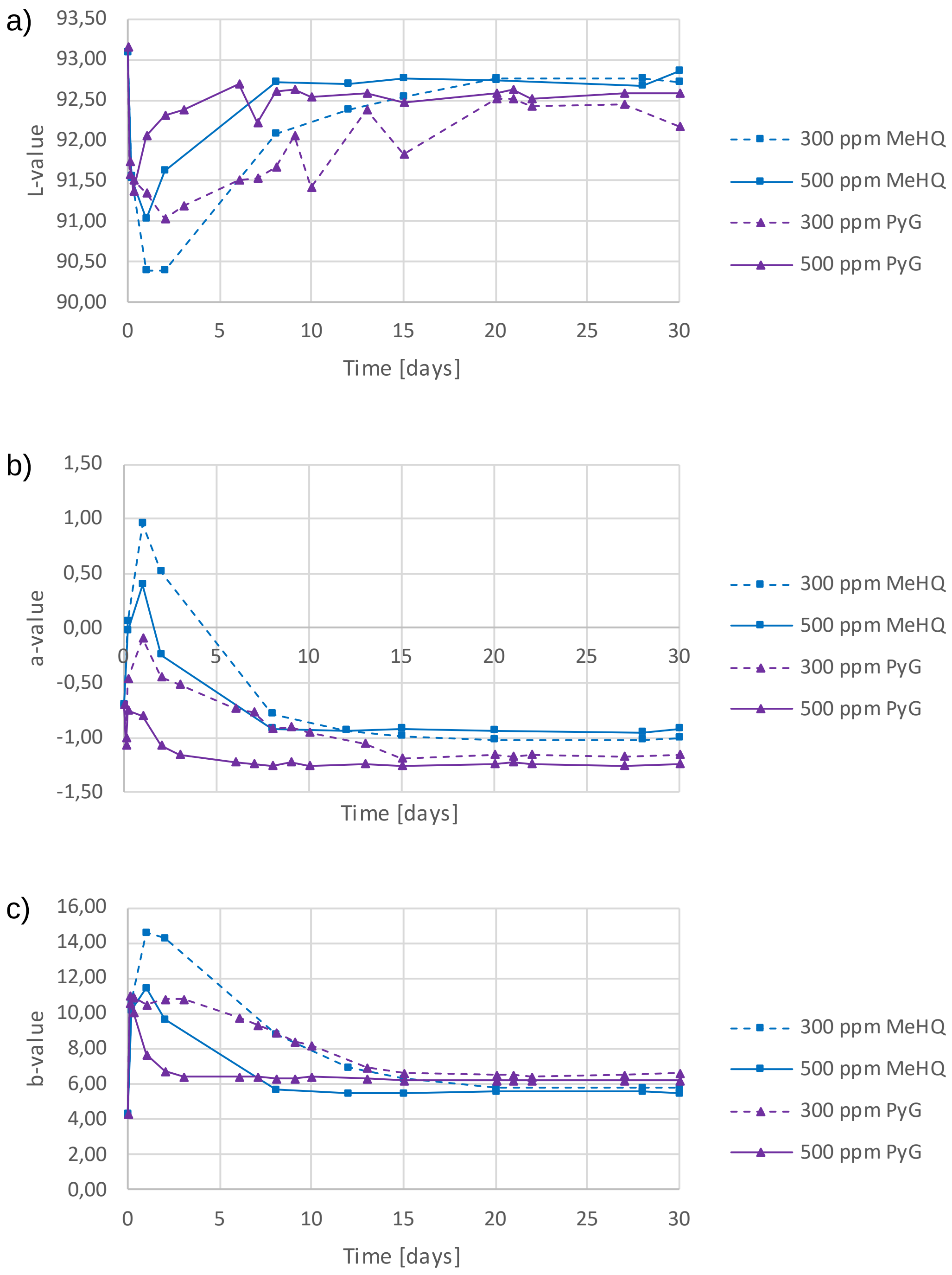

Figure S13: L-values (**a**), a-values (**b**), and b-values (**c**) of samples consisting of UDMA (without stabilizer), TPO (1 wt% ≙ 1.35 mol%), and 300/500 ppm MeHQ /PyG, stored at 50 °C immersed in water.

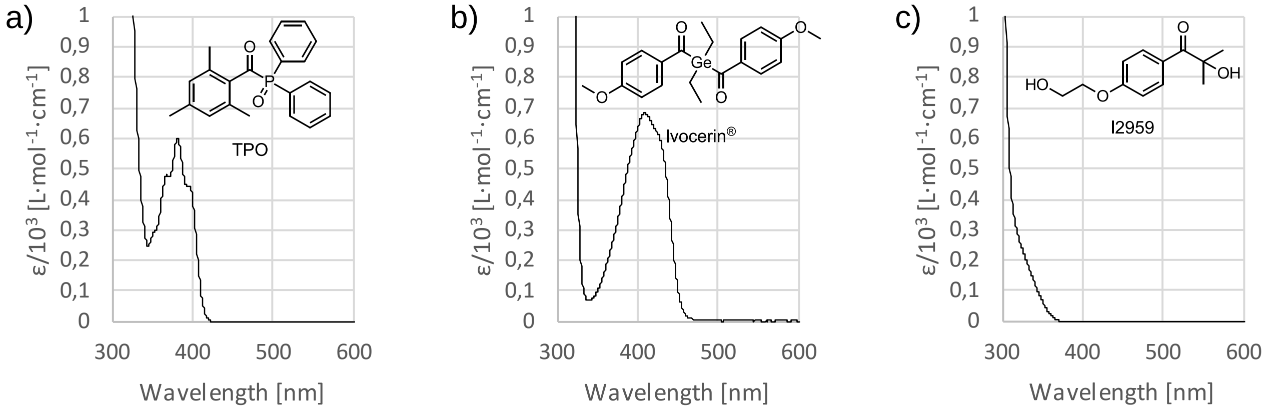

Figure S14: UV-Vis absorption spectrum of the photoinitiators TPO (**a**), Ivocerin® (**b**), and I2959 (**c**) in acetonitrile solution.

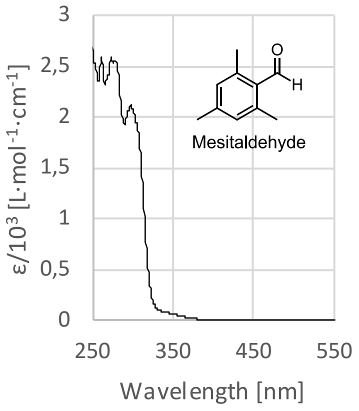

Figure S15: UV-Vis absorption spectrum of mestitaldehyde in acetonitrile solution.

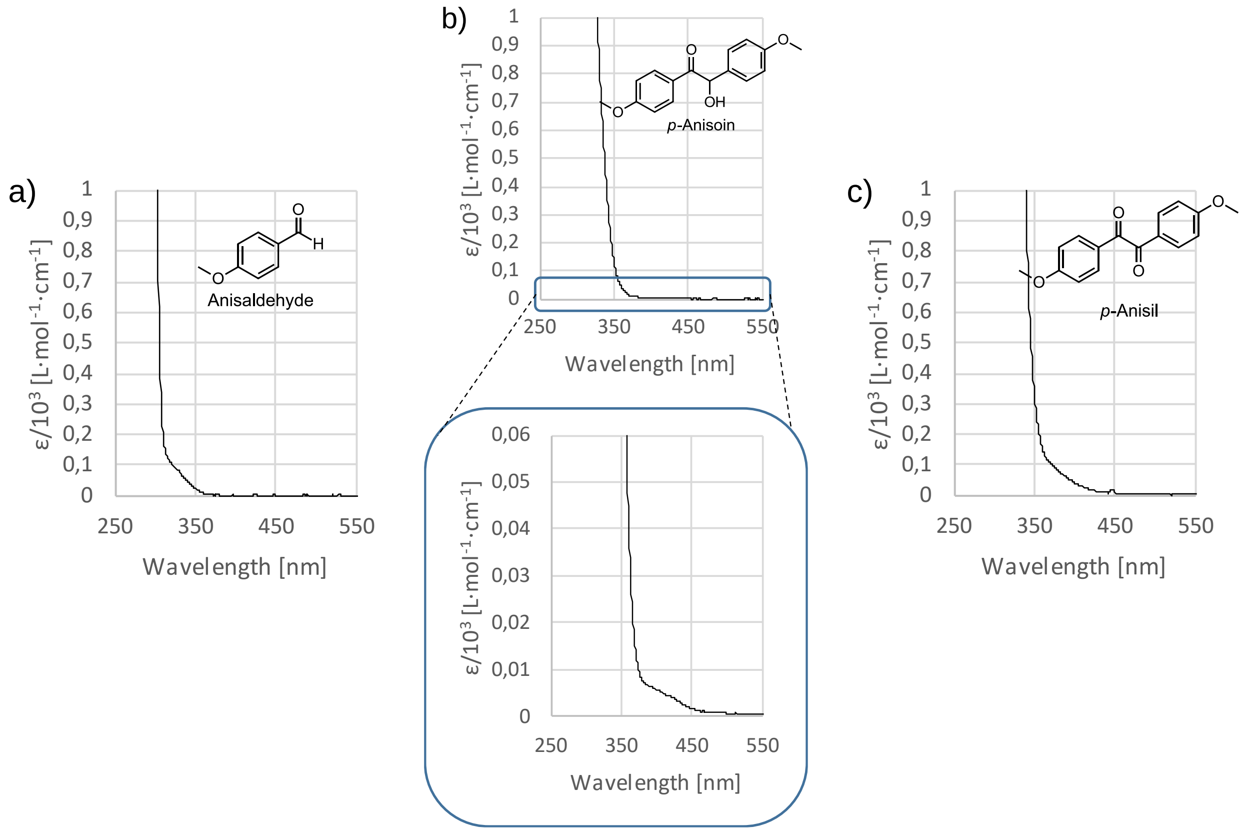

Figure S16: UV-Vis absorption spectra of anisaldehyde (**a**), p-anisoin (**b**), and p-anisil (**c**) in acetonitrile solution.

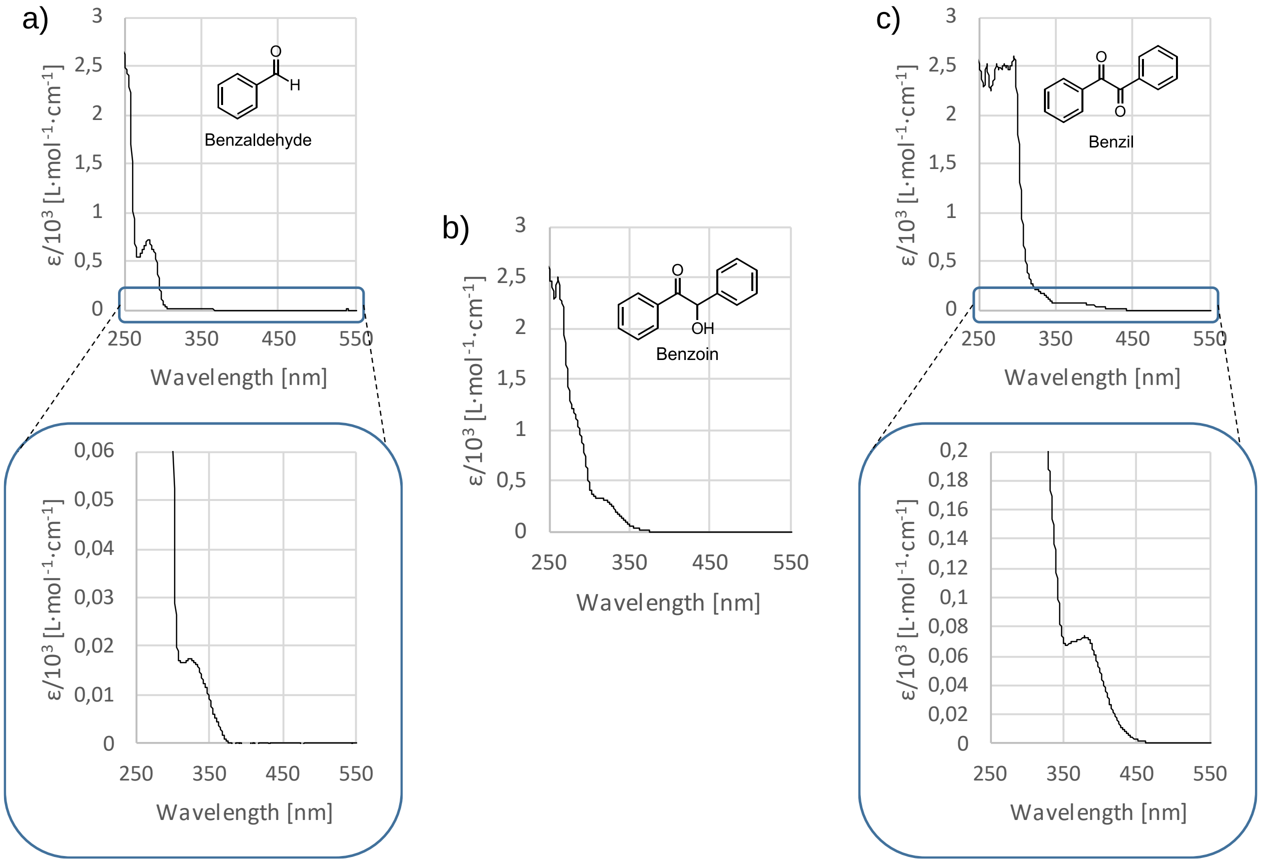

Figure S17: UV-Vis absorption spectra of benzaldehyd (**a**), benzoin (**b**), and benzill (**c**) in acetonitrile solution.
